# A non-manufacturer-sponsored, retrospective study to assess 2-year safety outcomes of the BellaGel^®^ SmoothFine as compared with its competitors in the context of the first Korean case of a medical device fraud

**DOI:** 10.1371/journal.pone.0259825

**Published:** 2023-02-02

**Authors:** Sang Eun Nam, Sangdal Lee, Younghye Cho, Jae Hong Kim

**Affiliations:** 1 Department of Surgery, Konkuk University Medical Center, Konkuk University School of Medicine, Seoul, Korea; 2 MD Clinic, Breast Center, Seoul, Korea; 3 Department of Pathology, Jangwon Medical Foundation, Seoul, Korea; 4 The W Clinic, Seoul, Korea; PLOS, UNITED KINGDOM

## Abstract

**Background:**

We conducted this study to assess preliminary 2-year safety outcomes of an implant-based augmentation mammaplasty using the BellaGel^®^ SmoothFine in the context of the first Korean case of a medical device fraud.

**Methods:**

Our clinical series of the patients (n = 579; 1,158 breasts) received augmentation using the BellaGel^®^ SmoothFine, Naturgel™, Motiva Ergonomix™, Eurosilicone Round Collection™, Natrelle^®^ INSPIRA™, Natrelle^®^ 410, Mentor^®^ MemoryGel Xtra or Microthane^®^. The patients were evaluated for incidences of postoperative complications and Kaplan-Meier survival and hazards.

**Results:**

Overall, there were a total of 101 cases (17.4%) of postoperative complications; these include 31 cases (5.4%) of shape deformity, 21 cases (3.6%) of CC, 18 cases (3.1%) of early seroma, 8 cases (1.4%) of infection, 5 cases (0.9%) of early hematoma, 1 case (0.2%) of delayed hematoma, 1 case (0.2%) of rupture and 1 case (0.2%) of ripping. Moreover, there were also 15 cases (2.6%) of other complications. There were significant differences in incidences of postoperative complications between the breast implants from different manufacturers (P = 0.034). The Natrelle^®^ 410 showed the longest survival (333.3±268.2 [141.5–525.1] days). A subgroup analysis showed that there were no significant differences in incidences of postoperative complications between the breast implants (P = 0.831). Moreover, the Natrelle^®^ INSPIRA™ showed the longest survival (223.7±107.1 [-42.3–489.6] days).

**Conclusions:**

Here, we describe preliminary 2-year safety outcomes of an implant-based augmentation mammaplasty using the BellaGel^®^ SmoothFine in the context of the first Korean case of a medical device fraud.

## Introduction

Great advances have been made for augmentation mammaplasty, accompanied by technological advancements in breast implants, which has led to an increase in implant-based augmentation mammaplasty [1]. According to the 2019 American Society of Plastic Surgeons (ASPS) statistics, a total of 299,715 patients underwent augmentation mammaplasty, 85% of whom received a silicone gel-filled breast implant [2]. Still, however, controversial opinions exist regarding the safety of silicone gel-filled breast implants. In more detail, there has been an extensive debate on the safety of breast implants, classified as a type IV medical device, since they were first introduced in the 1960s; their use in cosmetic surgery was transiently prohibited by the US Food and Drug Administration (FDA) between 1992 and 2006, but it was approved by the FDA in 2006 on condition that it would be under a long-term follow up, further analyzed after explanted and released with more detailed labeling [3, 4]. Nevertheless, a causal relationship of silicone gel-filled breast implants with postoperative complications, such as cancer, autoimmune disease and connective tissue diseases, has not been established, as described by a systematic review and meta-analyses of previous published studies in this series [5, 6]. Concerns for possible health risks after augmentation mammaplasty arose from earlier case reports about women exhibiting connective tissue disease after receiving breast implants or silicone injections [7, 8]. Such a key public health issue should be answered with scientific efforts rather than anti-scientific and irrational methods; it should be handled with evidence-based scientific grounds [9].

In Korea in July of 2007, a silicone gel-filled breast implant was first approved for clinical use by the Korean Ministry of Food and Drug Safety (KMFDS). Then, the Replicon^®^ (Polytech Health and Aesthetics, Dieburg, Germany), the Natrelle^®^ 410 (Allergan Inc., Irvine, CA) and the Naturgel™ (Groupe Sebbin SAS, Boissy-l’Aillerie, France) became commercially available in the early 2012. This was followed by the KMFDS approval of the Mentor^®^ CPG™ (Mentor Worldwide LLC, Santa Barbara, CA) and the Silimed (Sientra Inc., Santa Barbara, CA) in 2013. The BellaGel^®^ (HansBiomed Co. Ltd., Seoul, Korea), the only device from a Korean manufacturer, was released in November 27, 2015 [10].

Between 2016 and 2017, a silicone gel-filled breast implant with a microtextured surface was introduced to the Korean market. In June 17, 2016, the Motiva Ergonomix^TM^ (Establishment Labs Holdings Inc., Alajuela, Costa Rica) emerged in the Korean market and thereby initiated the era of a microtextured silicone gel-filled breast implant. In July 19, 2017, the BellaGel^®^ SmoothFine (formerly BellaGel^®^ Micro) (HansBiomed Co. Ltd.) became commercially available [11]. In August of 2018, the Mentor^®^ MemoryGel Xtra (Mentor Worldwide LLC) joined the group of silicone gel-filled breast implants in the Korean market [12].

Between 2007 and 2018, before the onset of the first Korean case of breast implant-associated anaplastic large cell lymphoma (BIA-ALCL), 222,470 textured breast implants had been circulated in the Korean market. Of these, 114,365 textured devices were the Allergan products and only 4,560 were from a Korean manufacturer, the HansBiomed Co. Ltd. Due to concerns for a causal relationship between a textured breast implant and the occurrence of BIA-ALCL, a textured device cannot be further used in Korea, as mandated by the KMFDS [13].

The global breast implant industry has faced a crisis, thus termed as a breast implant crisis. A breast implant crisis was further classified into the first crisis (Dow Corning), the second crisis (Poly Implant Prothèse [PIP]) and the third crisis (BIA-ALCL) [14, 15].

Recently in Korea, plastic surgeons, manufacturers of silicone gel-filled breast implant and patients have also faced a breast implant crisis [10, 11, 13]. Between 2019 and 2020 (August 16 and December 24, 2019 and October 5, 2020), a total of 3 cases of BIA-ALCL occurred in Korea. This warned stakeholders in the breast implant industry in Korea that they would face a crisis due to an implant failure. Indeed, the clinical use of textured breast implants was banned by the KMFDS in August 29, 2019. Then, they were forced to respond to a question regarding a possible causal relationship between a microtextured surface of the device and a risk of BIA-ALCL in Korea [11]. As reported by Kim JH, however, the first Korean case of a medical device fraud was committed by the manufacturer of the BellaGel^®^ implants including the BellaGel^®^ SmoothFine. According to the news media, a Korean manufacturer, the HansBiomed Co. Ltd., was investigated by the Korean police for using unapproved substances, such as 7–9700 and Q7-4850, and deliberately modifying the shell structure from 5 to 4 layers during the manufacturing process [10, 11].

To date, attempts have been made to compare the safety between the breast implants from different manufacturers in Korea [16, 17]. That is, Nam SY, et al. compared the vulnerability to capsular contracture (CC) based on surface properties between the BellaGel^®^ implants, including the BellaGel^®^ SmoothFine, and the Motiva Ergonomix^TM^ SilkSurface. These authors drew conclusions that the BellaGel^®^ SmoothFine was the least vulnerable to CC of the sample devices [16]. Moreover, Yoon S and Chang JH compared 1-year safety outcomes between the devices from 6 different manufacturers, thus reporting that the BellaGel^®^ SmoothFine is not inferior to its competitors [17].

Given the above background, we conducted this study to assess preliminary 2-year safety outcomes of an implant-based augmentation mammaplasty using the BellaGel^®^ SmoothFine as compared with its competitors in the Korean market in the context of the first Korean case of a medical device fraud.

## Patients and methods

### Study patients and setting

The current multi-center, retrospective, observational study was conducted in a total of 715 patients (n = 715) who underwent augmentation mammaplasty using breast implants at our hospitals between September 26, 2017 and October 21, 2019.

Inclusion criteria for the current study are as follows:

1. The patients who received augmentation mammaplasty using prosthetic implants for aesthetic or reconstructive purposes
2. The patients with available medical records.

Exclusion criteria for the current study are as follows:

1. The patients with missing values (n = 0)
2. The patients lost to follow-up (n = 136).

We therefore evaluated a total of 579 patients (n = 579) in the current study.

### Ethics statement

The current study was approved by the Internal Institutional Review Board of the Korea National Institute of Bioethics Policy; it was conducted in compliance with the relevant ethics guidelines. Written informed consent was waived due to its retrospective nature.

### Surgical procedures and postoperative monitoring of the patients

Surgical procedures were performed, as previously described [18, 19].

Postoperative course was meticulously monitored using a high-resolution ultrasound (HRUS) (Aplio i600; Canon Medical System, Otawara, Tochigi, Japan) during a regular follow-up at 1, 2, 3 and 4 weeks; 3, 6, 9 and 12 months; and thereafter [13].

### Patient evaluation and criteria

In the current study, the patients’ characteristics and survival of breast implants were analyzed, as previously described [18, 19]. We evaluated all the eligible patients (n = 579) depending on their baseline characteristics and complications. We also stratified them according to the age group (20–29, 30–39, 40–49, 50–59 and ≥60 years old), the length of follow-up period (<1 year and ≥1 year) and the trade name of breast implants. We classified breast implants as microtextured, round smooth and anatomical devices according to their shape and surface topography [20]. Then, we performed a comparative analysis of incidences of postoperative complications, cumulative complication survival and cumulative hazards. Postoperative complications were classified into implant-related and surgery-related ones.

Considering risk factors of developing complications of an implant-based augmentation mammaplasty, we performed a subgroup analysis of the patients’ postoperative complications. According to some “Core” studies, incidences of CC were significantly higher in secondary cases, such as revision surgery or reoperation, as compared with primary ones [21–24]. Moreover, it has been reported that there is a significant correlation between the use of anatomical implants and lower incidences CC [25]. Furthermore, locations of the implant pocket also serve as risk factors of developing CC; a subglandular pocket is commonly associated with higher incidences of CC as compared with a submuscular or dual-plane one [26]. We therefore excluded such factors as secondary cases, anatomical devices and subglandular pocket in comparing incidences of postoperative complications between the subgroups.

### Statistical analysis of the patient data

Values were expressed as the number of the patients with percentage, mean±SD (SD: standard deviation) or mean±SE (SE: standard error), where appropriate. Continuous variables were analyzed using the repeated measures analysis of variance (ANOVA), the Kruskal-Wallis test or Fisher’s exact test. Non-continuous variables were analyzed using the χ^2^-test. The cumulative overall complication-free survival was estimated, for which 95% confidence intervals (CIs) were provided. Moreover, differences in complication-free survival between the breast implants were tested for statistical significance using the repeated measures analysis of variance (ANOVA) and Duncan’s *post-hoc* analysis. Furthermore, the corresponding Kaplan-Meier survival and hazards were plotted as a curve. Statistical analysis was done using the SPSS ver. 18.0 for windows (SPSS Inc., Chicago, IL). A P-value of <0.05 was considered statistically significant.

## Results

### Baseline characteristics of the patients

**Fig 1:**
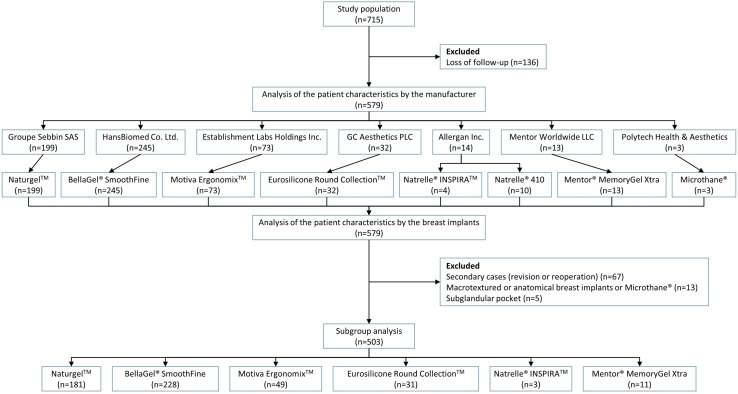
Disposition of the study patients.

The study population comprises a total of 715 patients (n = 715) who received an implant-based augmentation mammaplasty at our hospitals, 579 of whom met inclusion/exclusion criteria. All the eligible patients were Korean women with a mean age of 33.1±8.1 years old, who were followed up during a mean period of 218.4±153.2 days. Our clinical series of the patients include 578 bilateral cases and 1 unilateral case. We therefore evaluated a total of 1,157 breasts (578 left breasts and 579 right breasts). Baseline characteristics of the patients are represented in Table 1. Of these patients, a total of 503 (n = 503) were evaluated on a subgroup analysis. Disposition of the study patients is shown in Fig 1.

**Table 1:** Baseline characteristics of the patients (n = 579).

Variables		Values
**Age (years old)**		33.1±8.1 (20–65)
	20–29	261 (45.1%)
	30–39	190 (32.8%)
	40–49	101 (17.4%)
	50–59	22 (3.8%)
	≥60	3 (0.9%)
**Height (cm)**		162.5±5.0
**Weight (kg)**		51.2±5.7
**Follow-up period (days)**		218.4±153.2 (28–728)
	<1 year	448 (77.4%)
	≥1 year	131 (22.6%)
**Purpose of surgery**		
Left breast		
	Aesthetic augmentation mammaplasty	575 (99.3%)
	Reconstructive augmentation mammaplasty	3 (0.7%)
Right breast		
	Aesthetic augmentation mammaplasty	577 (99.7%)
	Reconstructive augmentation mammaplasty	2 (0.3%)
**Round of surgery**		
Left breast		
	Primary augmentation mammaplasty	511 (88.4%)
	Secondary augmentation mammaplasty	67 (11.6%)
Right breast		
	Primary augmentation mammaplasty	512 (88.4%)
	Secondary augmentation mammaplasty	67 (11.6%)
**Mode of incision**		
Left breast		
	Trans-axillary incision	542 (93.8%)
	Inframammary fold incision	22 (3.8%)
	Peri-areolar incision	8 (1.4%)
	Others	6 (1.0%)
Right side		
	Trans-axillary incision	542 (93.6%)
	Inframammary fold incision	24 (4.1%)
	Peri-areolar incision	8 (1.4%)
	Others	5 (0.9%)
**Type of pocket**		
Left breast		
	Subpectoral pocket	573 (99.1%)
	Subglandular pocket	5 (0.9%)
Right breast		
	Subpectoral pocket	574 (99.1%)
	Subglandular pocket	5 (0.9%)
**Volume of breast implant (cc)**		
Left breast		
	≤245	26 (4.5%)
	250–295	155 (26.8%)
	300–345	276 (47.8%)
	350–395	103 (17.8%)
	≥400	18 (3.1%)
Right breast		
	≤245	13 (2.2%)
	250–295	104 (18.0%)
	300–345	283 (48.9%)
	350–395	150 (25.9%)
	≥400	29 (5.0%)
**Shape of breast implant**		
Left breast		
	Round	565 (97.8%)
	Anatomical	13 (2.2%)
Right breast		
	Round	566 (97.8%)
	Anatomical	13 (2.2%)
**Surface topography of breast implant**		
Left breast		
	Macrotextured	10 (1.7%)
	Microtextured	548 (94.9%)
	Smooth	17 (2.9%)
	Microthane^®^	3 (0.5%)
Right breast		
	Macrotextured	10 (1.7%)
	Microtextured	549 (94.9%)
	Smooth	17 (2.9%)
	Microthane^®^	3 (0.5%)
**Profile of breast implant**		
Left breast		
	High	524 (90.7%)
	Medium	54 (9.3%)
	Low	0 (0.0%)
Right breast		
	High	543 (93.8%)
	Medium	36 (6.2%)
	Low	0 (0.0%)

Values are mean±standard deviation or the number of cases with percentage, where appropriate.

Of a total of 715 patients, 579 (n = 579) met inclusion/exclusion criteria. There were 67 patients (n = 67) receiving revision or reoperation, 13 (n = 13) receiving anatomical or textured breast implants and 5 (n = 5) receiving a device in the subglandular pocket. But 4 patients (n = 4) received revision or reoperation using anatomical or textured breast implants and 5 (n = 5) received revision or reoperation using a device in the subglandular pocket. A total of 503 patients (n = 503) were therefore evaluated on subgroup analysis.

**Fig 2:**
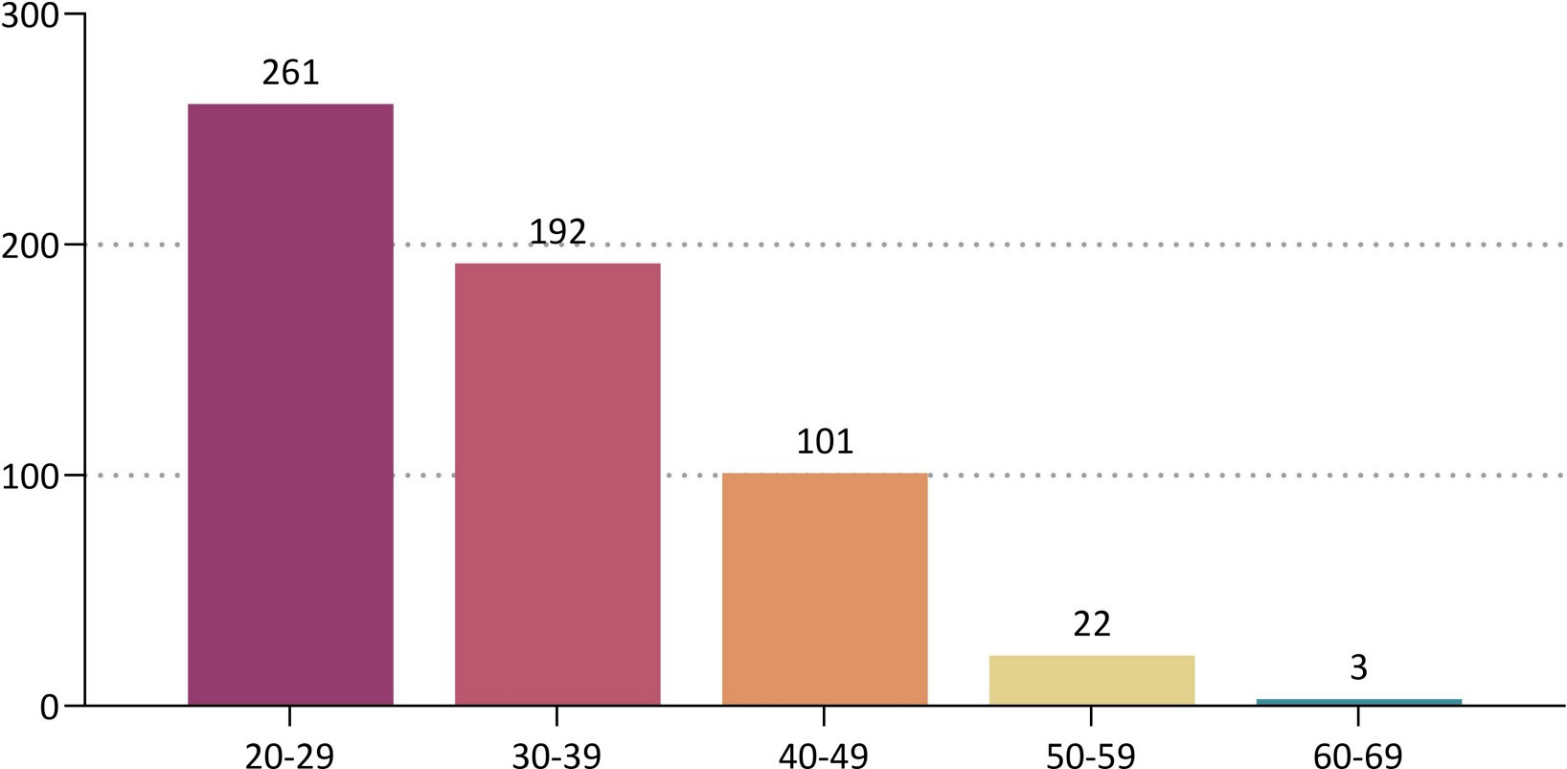
Distribution of the patients by the age group.

**Fig 3:**
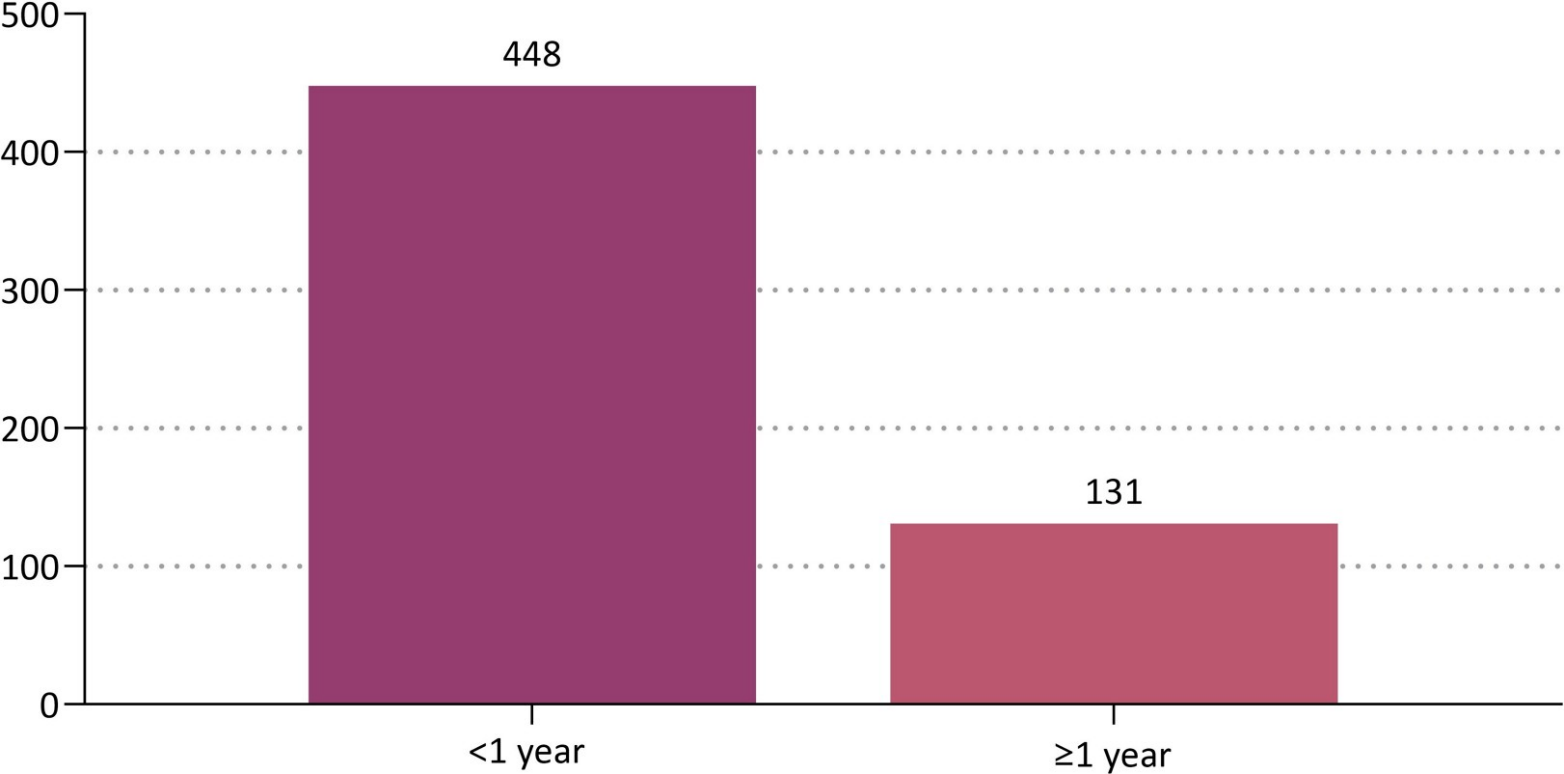
Distribution of the patients by the length of follow-up period based on a cut-off value of 1 year.

**Fig 4:**
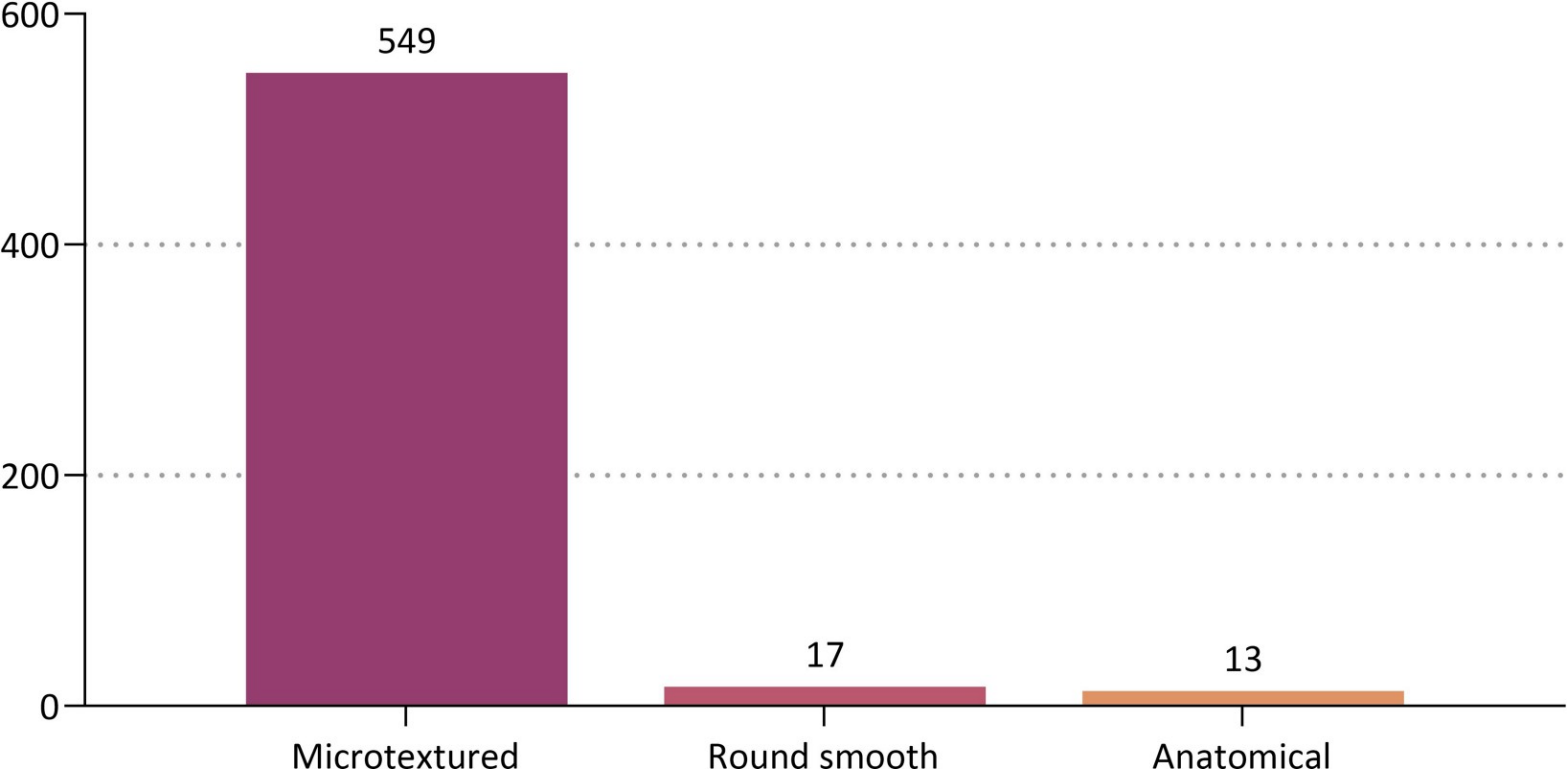
Distribution of the patients by the surface topography of breast implants.

By the age group, the patients aged between 20 and 29 years old were the most prevalent (45.1%, 261/579) (Table 2 and Fig 2). Based on a cut-off value of 1 year, 448 (77.4%) and 131 patients (22.6%) were followed up for <1 year and ≥1 year respectively (Table 3 and Fig 3). By the surface topography, the proportion of the patients receiving microtextured, round smooth and anatomical devices was 94.8% (549/579), 2.9% (17/579) and 2.2% (13/579), respectively (Fig 4).

**Table 2:** Baseline characteristics of the patients by the age group (n = 579).

Variables		Values				
		20–29 (n = 261)	30–39 (n = 192)	40–49 (n = 101)	50–59 (n = 22)	≥60 (n = 3)
**Age (years old)**		26.5±2.1 (20–29)	33.7±2.7 (30–39)	43.7±3.0 (40–49)	52.8±2.7 (50–58)	62.3±2.5 (60–65)
**Height (cm)**		162.2±4.8	163.0±5.1	163.4±4.7	159.3±4.1	160.0±4.6
**Weight (kg)**		50.2±5.7	51.7±5.8	52.6±5.4	52.0±5.7	50.3±3.2
**Follow-up period (days)**		217.0±158.8 (28–728)	212.8±147.7 (41–601)	230.8±154.1 (55–575)	233.5±134.4 (79–543)	173.0±149.0 (82–345)
**Purpose of surgery**						
Left breast						
	Aesthetic augmentation mammaplasty	261 (100.0%)	192 (95.5%)	99 (98.0%)	20 (90.9%)	3 (100.0%)
	Reconstructive augmentation mammaplasty	0 (0.0%)	0 (0.0%)	1 (1.0%)	2 (9.1%)	0 (0.0%)
Right breast						
	Aesthetic augmentation mammaplasty	261 (100.0%)	192 (100.0%)	99 (98.0%)	22 (100.0%)	3 (100.0%)
	Reconstructive augmentation mammaplasty	0 (0.0%)	0 (0.0%)	2 (2.0%)	0 (0.0%)	0 (0.0%)
**Round of surgery**						
Left breast						
	Primary augmentation mammaplasty	254 (97.3%)	172 (89.6%)	74 (74.3%)	11 (50.0%)	0 (0.0%)
	Secondary augmentation mammaplasty	7 (2.7%)	20 (10.4%)	26 (25.7%)	11 (50.0%)	3 (100.0%)
Right breast						
	Primary augmentation mammaplasty	254 (97.3%)	172 (89.6%)	75 (74.3%)	11 (50.0%)	0 (0.0%)
	Secondary augmentation mammaplasty	7 (2.7%)	20 (10.4%)	26 (25.7%)	11 (50.0%)	3 (100.0%)
**Mode of incision**						
Left breast						
	Trans-axillary incision	256 (98.1%)	179 (93.1%)	89 (89.0%)	16 (72.7%)	2 (66.7%)
	Inframammary fold incision	4 (1.5%)	8 (4.2%)	7 (7.0%)	2 (9.1%)	1 (33.3%)
	Peri-areolar incision	1 (0.4%)	2 (1.1%)	2 (2.0%)	3 (13.6%)	0 (0.0%)
	Others	0 (0.0%)	3 (1.6%)	2 (2.0%)	1 (4.6%)	0 (0.0%)
Right breast						
	Trans-axillary incision	256 (98.1%)	179 (93.1%)	89 (88.1%)	16 (72.8%)	2 (66.7%)
	Inframammary fold incision	4 (1.5%)	8 (4.2%)	8 (7.9%)	3 (13.6%)	1 (33.3%)
	Peri-areolar incision	1 (0.4%)	2 (1.1%)	2 (2.0%)	3 (13.6%)	0 (0.0%)
	Others	0 (0.0%)	3 (1.6%)	2 (2.0%)	0 (0.0%)	0 (0.0%)
**Type of pocket**						
Left breast						
	Subpectoral pocket	260 (99.6%)	190 (98.9%)	99 (99.0%)	21 (95.4%)	3 (100.0%)
	Subglandular pocket	1 (0.4%)	2 (1.1%)	1 (1.0%)	1 (4.6%)	0 (0.0%)
Right breast						
	Subpectoral pocket	260 (99.6%)	190 (98.9%)	100 (99.0%)	21 (95.4%)	3 (100.0%)
	Subglandular pocket	1 (0.4%)	2 (1.1%)	1 (1.0%)	1 (4.6%)	0 (0.0%)
**Volume of breast implant (cc)**						
Left breast						
	≤245	16 (6.1%)	3 (1.6%)	3 (3.0%)	3 (13.6%)	1 (33.3%)
	250–295	77 (29.8%)	49 (25.1%)	24 (24.0%)	4 (18.2%)	1 (33.3%)
	300–345	125 (47.7%)	90 (47.2%)	48 (48.0%)	12 (54.5%)	1 (33.3%)
	350–395	39 (14.9%)	44 (23.0%)	19 (19.0%)	1 (4.6%)	0 (0.0%)
	≥400	4 (1.5%)	6 (3.1%)	6 (6.0%)	2 (9.1%)	0 (0.0%)
Right breast						
	≤245	5 (1.9%)	1 (0.5%)	1 (1.0%)	6 (27.3%)	0 (0.0%)
	250–295	53 (20.3%)	23 (12.0%)	22 (21.7%)	4 (18.2%)	2 (66.7%)
	300–345	131 (50.2%)	100 (52.1%)	42 (41.6%)	9 (40.8%)	1 (33.3%)
	350–395	63 (24.1%)	54 (28.1%)	31 (30.7%)	2 (9.1%)	0 (0.0%)
	≥400	9 (3.5%)	14 (7.3%)	5 (5.0%)	1 (4.6%)	0 (0.0%)
**Shape of breast implant**						
Left breast						
	Round	257 (98.5%)	188 (97.9%)	97 (97.0%)	20 (90.9%)	3 (100.0%)
	Anatomical	4 (1.5%)	4 (2.1%)	3 (3.0%)	2 (9.1%)	0 (0.0%)
Right breast						
	Round	257 (98.5%)	188 (97.9%)	98 (97.0%)	20 (90.9%)	3 (100.0%)
	Anatomical	4 (1.5%)	4 (2.1%)	3 (3.0%)	2 (9.1%)	0 (0.0%)
**Surface topography of breast implant**						
Left breast						
	Macrotextured	4 (1.5%)	4 (2.1%)	1 (1.0%)	1 (4.6%)	0 (0.0%)
	Microtextured	251 (96.2%)	181 (94.2%)	94 (94.0%)	19 (86.2%)	3 (100.0%)
	Smooth	6 (2.3%)	7 (3.7%)	3 (3.0%)	1 (4.6%)	0 (0.0%)
	Microthane^®^	0 (0.0	0 (0.0	2 (2.0)	1 (4.6)	0 (0.0
Right breast						
	Macrotextured	4 (1.5%)	4 (2.1%)	1 (1.0%)	1 (4.6%)	0 (0.0%)
	Microtextured	251 (96.2%)	181 (94.2%)	95 (94.0%)	19 (86.2%)	3 (100.0%)
	Smooth	6 (2.3%)	7 (3.7%)	3 (3.0%)	1 (4.6%)	0 (0.0%)
	Microthane^®^	0 (0.0%)	0 (0.0%)	2 (2.0%)	1 (4.6%)	0 (0.0%)
**Profile of breast implant**						
Left breast						
	High	239 (91.6%)	179 (93.2%)	85 (85.0%)	19 (86.4%)	2 (66.7%)
	Medium	22 (8.4%)	13 (6.8%)	15 (15.0%)	3 (13.6%)	1 (33.3%)
	Low	0 (0.0%)	0 (0.0%)	0 (0.0%)	0 (0.0%)	0 (0.0%)
Right breast						
	High	249 (95.4%)	188 (97.4%)	87 (86.1%)	18 (81.8%)	2 (66.7%)
	Medium	12 (4.6%)	5 (2.6%)	14 (13.9%)	4 (18.2%)	1 (33.3%)
	Low	0 (0.0%)	0 (0.0%)	0 (0.0%)	0 (0.0%)	0 (0.0%)

Values are mean±standard deviation or the number of cases with percentage, where appropriate.

**Table 3:** Baseline characteristics of the patients by the length of follow-up period based on a cut-off value of 1 year (n = 579).

Variables		Values	
		FU period <1 year (n = 448)	FU period ≥1 year (n = 131)
**Age (years old)**		33.0±8.2 (20–65)	33.4±7.5 (22–52)
**Height (cm)**		162.4±4.9	163.1±4.9
**Weight (kg)**		51.2±5.8	51.1±5.7
**FU period (days)**		150.1±89.4 (28–365)	452.1±75.5 (366–728)
**Purpose of surgery**			
Left breast			
	Aesthetic augmentation mammaplasty	445 (99.3%)	130 (99.2%)
	Reconstructive augmentation mammaplasty	3 (0.7%)	0 (0.0%)
Right breast			
	Aesthetic augmentation mammaplasty	447 (99.8%)	130 (99.2%)
	Reconstructive augmentation mammaplasty	1 (0.2%)	1 (0.8%)
**Round of surgery**			
Left breast			
	Primary augmentation mammaplasty	398 (88.8%)	113 (86.3%)
	Secondary augmentation mammaplasty	50 (11.2%)	17 (13.0%)
Right breast			
	Primary augmentation mammaplasty	398 (88.8%)	114 (87.0%)
	Secondary augmentation mammaplasty	50 (11.2%)	17 (13.0%)
**Mode of incision**			
Left breast			
	Trans-axillary incision	418 (93.3%)	124 (95.4%)
	Inframammary fold incision	16 (3.6%)	6 (4.6%)
	Peri-areolar incision	8 (1.8%)	0 (0.0%)
	Others	6 (1.3%)	0 (0.0%)
Right breast			
	Trans-axillary incision	418 (93.3%)	124 (94.7%)
	Inframammary fold incision	17 (3.8%)	7 (5.3%)
	Peri-areolar incision	8 (1.8%)	0 (0.0%)
	Others	5 (1.1%)	0 (0.0%)
**Type of pocket**			
Left breast			
	Subpectoral pocket	446 (99.5%)	127 (97.7%)
	Subglandular pocket	2 (0.5%)	3 (2.3%)
Right breast			
	Subpectoral pocket	446 (99.5%)	128 (97.7%)
	Subglandular pocket	2 (0.5%)	3 (2.3%)
**Volume of breast implant (cc)**			
Left breast			
	≤245	24 (5.4%)	2 (1.5%)
	250–295	140 (31.3%)	15 (11.5%)
	300–345	200 (44.6%)	76 (58.5%)
	350–395	70 (15.6%)	33 (25.4%)
	≥400	14 (3.1%)	4 (3.1%)
Right breast			
	≤245	12 (2.7%)	1 (0.8%)
	250–295	90 (20.1%)	14 (10.7%)
	300–345	220 (49.1%)	63 (48.1%)
	350–395	105 (23.4%)	45 (34.4%)
	≥400	21 (4.7%)	8 (6.1%)
**Shape of breast implant**			
Left breast			
	Round	438 (97.8%)	127 (97.7%)
	Anatomical	10 (2.2%)	3 (2.3%)
Right breast			
	Round	438 (97.8%)	128 (97.7%)
	Anatomical	10 (2.2%)	3 (2.3%)
**Surface topography of breast implant**			
Left breast			
	Macrotextured	7 (1.6%)	3 (2.3%)
	Microtextured	428 (95.5%)	127 (97.7%)
	Smooth	10 (2.2%)	0 (0.0%)
	Microthane^®^	3 (0.7%)	0 (0.0%)
Right breast			
	Macrotextured	7 (1.6%)	3 (2.3%)
	Microtextured	428 (95.5%)	128 (97.7%)
	Smooth	10 (2.2%)	0 (0.0%)
	Microthane^®^	3 (0.7%)	0 (0.0%)
**Profile of breast implant**			
Left breast			
	High	407 (90.9%)	117 (90.0%)
	Medium	41 (9.1%)	13 (10.0%)
	Low	0 (0.0%)	0 (0.0%)
Right breast			
	High	423 (94.4%)	120 (91.6%)
	Medium	25 (5.6%)	11 (8.4%)
	Low	0 (0.0%)	0 (0.0%)

**Abbreviations:** FU, follow-up.

Values are mean±standard deviation or the number of cases with percentage, where appropriate.

**Fig 5:**
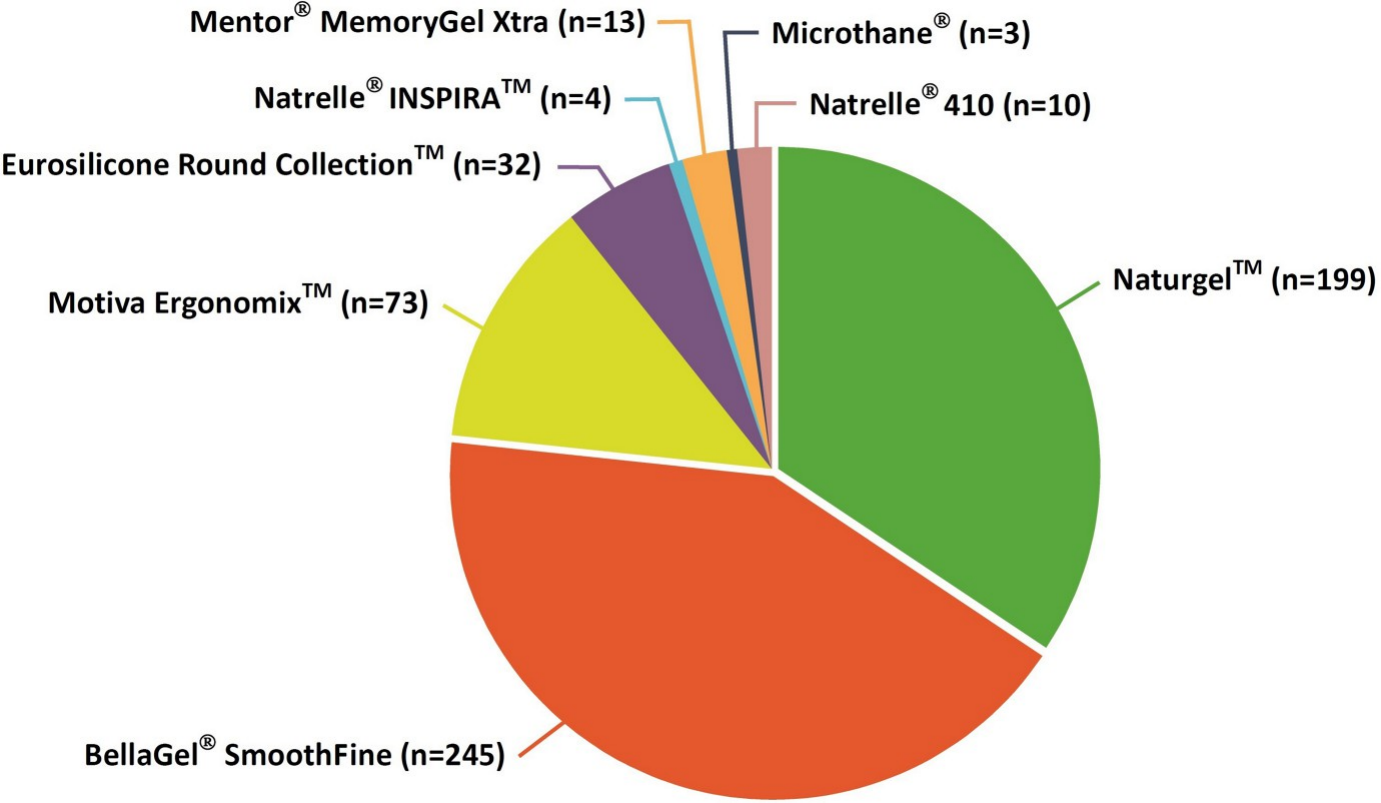
Distribution of the patients by the trade name of breast implants.

Our clinical series of the patients received augmentation mammaplasty using prosthetic breast implants, and these include the Naturgel™ (n = 199), the BellaGel^®^ SmoothFine (n = 245), the Motiva Ergonomix™ (n = 73), the Eurosilicone Round Collection™ (GC Aesthetics PLC, Apt Cedex, France) (n = 32), the Natrelle^®^ INSPIRA™ (Allergan Inc.) (n = 4), the Natrelle^®^ 410 (n = 10), the Mentor^®^ MemoryGel Xtra (n = 13) or the Microthane^®^ (n = 3) (Polytech Health & Aesthetics, Dieburg, Germany) (Fig 5). Baseline characteristics of the patients by the trade name of breast implants are represented in Table 4.

**Table 4:** Baseline characteristics of the patients by the trade name of breast implants (n = 579).

Variables		Values							
		Microtextured breast implants				Round smooth breast implants		Anatomical breast implants	
		Naturgel™ (n = 199)	BellaGel^®^ SmoothFine (n = 245)	Motiva Ergonomix™ (n = 73)	Eurosilicone Round Collection™ (n = 32)	Natrelle^®^ INSPIRA™ (n = 4)	Mentor^®^ MemoryGel Xtra (n = 13)	Microthane^®^ (n = 3)	Natrelle^®^ 410 (n = 10)
**Age (years old)**		32.7± 8.5 (20–65)	32.7±7.6 (21–58)	35.9±8.5 (22–56)	29.9±6.9 (21–48)	42.5±9.7 (34–54)	31.0±6.7 (20–44)	47.0±3.6 (44–51)	34.6±9.3 (27–57)
**Height (cm)**		162.4±4.8	162.6±5.0	163.6±5.0	161.2±5.4	164.0±6.1	161.5±4.0	159.7±2.5	165.5±5.4
**Weight (kg)**		51.3±5.7	50.8±5.4	52.2±5.6	50.6±7.0	60.5±11.7	50.5±5.6	42.0±1.7	52.0±6.0
**FU period (days)**		229.4±154.7 (41–624)	223.2±149.6 (28–612)	220.1±161.9 (50–639)	128.2±80.3 (79–415)	202.0±97.6 (105–313)	110.3±59.7 (80–300)	251.7±13.7 (237–364)	295.6±276.3 (81–728)
**Purpose of surgery**									
Left breast									
	Aesthetic augmentation mammaplasty	199 (100.0%)	243 (99.6%)	72 (98.6%)	32 (100.0%)	3 (75.0%)	13 (100.0%)	3 (100.0%)	10 (100.0%)
	Reconstructive augmentation mammaplasty	0 (0.0%)	1 (0.4%)	1 (0.4%)	0 (0.0%)	1 (25.0%)	0 (0.0%)	0 (0.0%)	0 (0.0%)
Right breast									
	Aesthetic augmentation mammaplasty	199 (100.0%)	244 (99.6%)	73 (100.0%)	31 (96.9%)	14 (100.0%)	13 (100.0%)	3 (100.0%)	0 (0.0%)
	Reconstructive augmentation mammaplasty	0 (0.0%)	1 (0.4%)	0 (0.0%)	1 (0.4%)	0 (0.0%)	0 (0.0%)	0 (0.0%)	0 (0.0%)
**Round of surgery**									
Left breast									
	Primary augmentation mammaplasty	181 (90.9%)	227(93.0%)	49 (67.1%)	31 (96.9%)	3 (75.0%)	11 (84.6%)	0 (0.0%)	9 (90.0%)
	Secondary augmentation mammaplasty	18 (9.1%)	17(7.0%)	24 (32.9%)	1 (3.1%)	1 (25.0%)	2 (15.4%)	3 (100.0%)	1 (10.0%)
Right breast									
	Primary augmentation mammaplasty	181 (91.0%)	228 (93.1%)	49 (67.1%)	31 (96.9%)	3 (75.0%)	11 (84.6%)	0 (0.0%)	9 (90.0%)
	Secondary augmentation mammaplasty	18 (9.1%)	17 (6.9%)	24 (32.9%)	1 (3.1%)	1 (25.0%)	2 (15.4%)	3 (100.0%)	1 (10.0%)
**Mode of incision**									
Left breast									
	Trans-axillary incision	184 (92.5%)	238 (97.6%)	63 (86.3%)	31 (96.9%)	2 (50.0%)	13 (100.0%)	2 (66.7%)	9 (90.0%)
	IMF incision	13 (6.5%)	3 (1.2%)	5 (6.9%)	1 (3.1%)	0 (0.0%)	0 (0.0%)	0 (0.0%)	0 (0.0%)
	Peri-areolar incision	2 (1.0%)	1 (0.4%)	3 (4.1%)	0 (0.0%)	0 (0.0%)	0 (0.0%)	1 (33.3%)	1 (10.0%)
	Others	0 (0.0%)	2 (0.8%)	2 (2.7%)	0 (0.0%)	2 (50.0%)	0 (0.0%)	0 (0.0%)	0 (0.0%)
Right breast									
	Trans-axillary incision	184 (92.5%)	238 (97.2%)	63 (86.3%)	31 (96.9%)	2 (50.0%)	13 (100.0%)	2 (66.7%)	9 (90.0%)
	IMF incision	13 (6.5%)	4 (1.6%)	5 (6.9%)	1 (3.1%)	1 (25.0%)	0 (0.0%)	0 (0.0%)	0 (0.0%)
	Peri-areolar incision	2 (1.0%)	1 (0.4%)	3 (4.1%)	0 (0.0%)	0 (0.0%)	0 (0.0%)	1 (33.3%)	1 (10%)
	Others	0 (0.0%)	2 (0.8%)	2 (2.7%)	0 (0.0%)	1 (25.0%)	0 (0.0%)	0 (0.0%)	0 (0.0%)
**Type of pocket**									
Left breast									
	Subpectoral pocket	199 (100.0%)	241 (98.8%)	71 (97.3%)	32 (100.0%)	4 (100.0%)	13 (100.0%)	3 (100.0%)	10 (100.0%)
	Subglandular pocket	0 (0.0%)	3 (1.2%)	0 (0.0%)	0 (0.0%)	0 (0.0%)	0 (0.0%)	0 (0.0%)	0 (0.0%)
Right breast									
	Subpectoral pocket	199 (100.0%)	242 (98.8%)	71 (97.3%)	32 (100.0%)	4 (100.0%)	13 (100.0%)	3 (100.0%)	10 (100.0%)
	Subglandular pocket	0 (0.0%)	3 (1.2%)	2 (2.7%)	0 (0.0%)	0 (0.0%)	0 (0.0%)	0 (0.0%)	0 (0.0%)
**Volume of breast implant (cc)**									
Left breast									
	≤245	9 (4.5%)	5 (2.1%)	3 (4.1%)	4 (12.5%)	0 (0.0%)	2 (15.3%)	2 (66.7%)	1 (10.0%)
	250–295	51 (25.7%)	61 (25.0%)	21 (28.8%)	13 (40.6%)	0 (0.0%)	5 (38.6%)	1 (33.3%)	3 (30.0%)
	300–345	97 (48.7%)	136 (55.7%)	25 (34.2%)	10 (31.3%)	1 (25.0%)	4 (30.8%)	0 (0.0%)	3 (30.0%)
	350–395	42 (21.1%)	35 (14.3%)	17 (23.3%)	4 (12.5%)	0 (0.0%)	2 (15.3%)	0 (0.0%)	3 (30.0%)
	≥400	0 (0.0%)	7 (2.9%)	7 (9.6%)	1 (3.1%)	3 (75.0%)	0 (0.0%)	0 (0.0%)	0 (0.0%)
Right breast									
	≤245	2 (1.0%)	4 (1.6%)	4 (5.5%)	1 (3.1%)	1 (25.0%)	0 (0.0%)	1 (33.3%)	0 (0.0%)
	250–295	29 (14.6%)	34 (13.9%)	19 (26.0%)	11 (34.4%)	0 (0.0%)	6 (46.1%)	1 (33.3%)	4 (40.0%)
	300–345	101 (50.7%)	138 (56.3%)	25 (34.3%)	11 (34.4%)	1 (25.0%)	3 (23.1%)	1 (33.3%)	3 (30.0%)
	350–395	67 (33.7%)	58 (23.7%)	12 (16.4%)	8 (25.0%)	0 (0.0%)	3 (23.1%)	0 (0.0%)	2 (20.0%)
	≥400	0 (0.0%)	11 (4.5%)	13 (17.8%)	1 (3.1%)	2 (50.0%)	1 (7.7%)	0 (0.0%)	1 (10.0%)
**Shape of breast implant**									
Left breast									
	Round	199 (100.0%)	244 (100.0%)	73 (100.0%)	32 (100.0%)	4 (100.0%)	13 (100.0%)	0 (0.0%)	0 (0.0%)
	Anatomical	0 (0.0%)	0 (0.0%)	0 (0.0%)	0 (0.0%)	0 (0.0%)	0 (0.0%)	3 (100.0%)	10 (100.0%)
Right breast									
	Round	199 (100.0%)	245 (100.0%)	73 (100.0%)	32 (100.0%)	4 (100.0%)	13 (100.0%)	0 (0.0%)	0 (0.0%)
	Anatomical	0 (0.0%)	0 (0.0%)	0 (0.0%)	0 (0.0%)	0 (0.0%)	0 (0.0%)	3 (100.0%)	10 (100.0%)
**Surface topography of breast implant**									
Left breast									
	Macrotextured	0 (0.0%)	0 (0.0%)	0 (0.0%)	0 (0.0%)	0 (0.0%)	0 (0.0%)	0 (0.0%)	10 (100.0%)
	Microtextured	199 (100.0%)	244 (100.0%)	73 (100.0%)	32 (100.0%)	0 (0.0%)	0 (0.0%)	0 (0.0%)	0 (0.0%)
	Smooth	0 (0.0%)	0 (0.0%)	0 (0.0%)	0 (0.0%)	4 (100.0%)	13 (100.0%)	0 (0.0%)	0 (0.0%)
	Microthane^®^	0 (0.0%)	0 (0.0%)	0 (0.0%)	0 (0.0%)	0 (0.0%)	0 (0.0%)	3 (100.0%)	0 (0.0%)
Right breast									
	Macrotextured	0 (0.0%)	0 (0.0%)	0 (0.0%)	0 (0.0%)	0 (0.0%)	0 (0.0%)	0 (0.0%)	10 (100.0%)
	Microtextured	199 (100.0%)	245 (100.0%)	73 (100.0%)	32 (100.0%)	0 (0.0%)	0 (0.0%)	0 (0.0%)	0 (0.0%)
	Smooth	0 (0.0%)	0 (0.0%)	0 (0.0%)	0 (0.0%)	4 (100.0%)	13 (100.0%)	0 (0.0%)	0 (0.0%)
	Microthane^®^	0 (0.0%)	0 (0.0%)	0 (0.0%)	0 (0.0%)	0 (0.0%)	0 (0.0%)	3 (100.0%)	0 (0.0%)
**Profile of breast implant**									
Left breast									
	High	179 (90.0%)	229 (93.9%)	61 (83.6%)	28 (87.5%)	4 (100.0%)	13 (100.0%)	0 (0.0%)	10 (100.0%)
	Medium	20 (10.0%)	15 (6.1%)	12 (16.4%)	4 (12.5%)	0 (0.0%)	0 (0.0%)	3 (100.0%)	0 (0.0%)
	Low	0 (0.0%)	0 (0.0%)	0 (0.0%)	0 (0.0%)	0 (0.0%)	0 (0.0%)	0 (0.0%)	0 (0.0%)
Right breast									
	High	182 (91.5%)	238 (97.1%)	65 (89.0%)	31 (96.9%)	4 (100.0%)	13 (100.0%)	0 (0.0%)	10 (100.0%)
	Medium	17 (8.5%)	7 (2.9%)	8 (11.0%)	1 (3.1%)	0 (0.0%)	0 (0.0%)	3 (100.0%)	0 (0.0%)
	Low	0 (0.0%)	0 (0.0%)	0 (0.0%)	0 (0.0%)	0 (0.0%)	0 (0.0%)	0 (0.0%)	0 (0.0%)

**Abbreviations:** FU, follow-up; IMF, inframammary fold.

Values are mean±standard deviation or the number of cases with percentage, where appropriate.

### Safety outcomes

**Fig 6:**
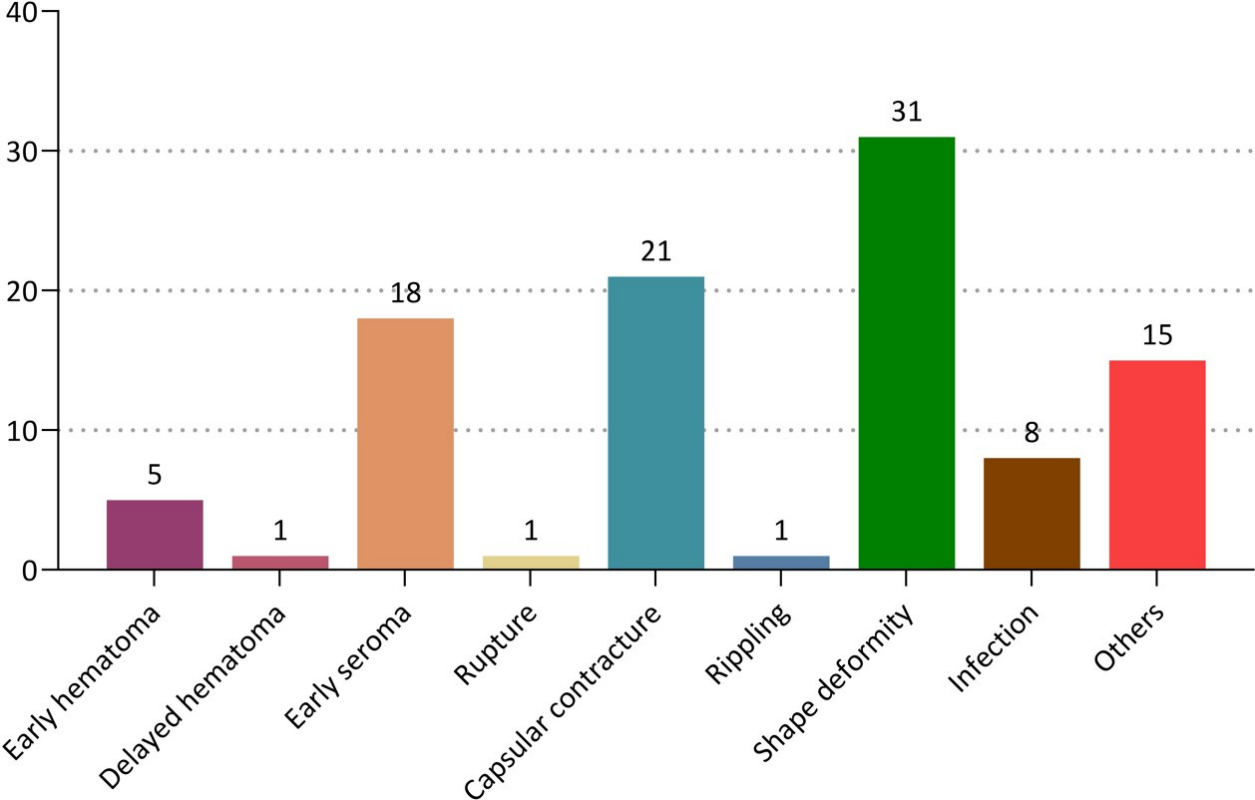
Incidences of postoperative complications.

Overall, there were a total of 101 cases (17.4%) of postoperative complications; these include 31 cases (5.4%) of shape deformity, 21 cases (3.6%) of CC, 18 cases (3.1%) of early seroma, 8 cases (1.4%) of infection, 5 cases (0.9%) of early hematoma, 1 case (0.2%) of delayed hematoma, 1 case (0.2%) of rupture and 1 case (0.2%) of ripping. Moreover, there were also 15 cases (2.6%) of other complications (Fig 6). Of these, early seroma, early hematoma and delayed hematoma correspond to surgery-related complications and shape deformity, CC, infection, rupture and rippling do implant-related complications.

As shown in Table 5, there were significant differences in incidences of postoperative complications between the age groups; there were age-dependent increases in them (P<0.05). But there were no significant differences in incidences of postoperative complications between the two groups divided based on a cut-off value of 1 year (P>0.05) (Table 6).

**Table 5:** Incidences of postoperative complications by the age group (n = 579).

Variables		Values					P
		20–29 (n = 261)	30–39 (n = 192)	40–49 (n = 101)	50–59 (n = 22)	≥60 (n = 3)	
**Total incidences**		34 (13.0%)	35 (18.4%)	21 (20.8%)	9 (40.9%)	2 (66.7%)	<0.001*
	Early hematoma	1 (0.4%)	1 (0.5%)	2 (2.0%)	1 (4.6%)	0 (0.0%)	
	Delayed hematoma	1 (0.4%)	0 (0.0%)	0 (0.0%)	0 (0.0%)	0 (0.0%)	
	Early seroma	5 (1.9%)	6 (3.1%)	6 (5.9%)	1 (4.6%)	0 (0.0%)	
	Rupture	0 (0.0%)	1 (0.5%)	0 (0.0%)	0 (0.0%)	0 (0.0%)	
	Capsular contracture	6 (2.3%)	8 (4.2%)	3 (3.0%)	3 (13.6%)	1 (33.3%)	
	Rippling	1 (0.4%)	0 (0.0%)	0 (0.0%)	0 (0.0%)	0 (0.0%)	
	Shape deformity	11 (4.2%)	8 (4.2%)	8 (7.9%)	3 (13.6%)	1 (33.3%)	
	Infection	4 (1.5%)	2 (1.1%)	2 (2.0%)	0 (0.0%)	0 (0.0%)	
	Others	5 (1.9%)	9 (4.7%)	0 (0.0%)	1 (4.6%)	0 (0.0%)	

Values are the number of cases with percentage.

*Statistical significance at P<0.05.

**Table 6:** Incidences of postoperative complications by the length of follow-up period based on a cut-off value of 1 year (n = 579).

Variables		Values		P
		FU period <1 year (n = 448)	FU period ≥1 year (n = 131)	
**Total incidences**		74 (16.5%)	27 (20.6%)	>0.05
	Early hematoma	3 (0.7%)	2 (1.5%)	
	Delayed hematoma	1 (0.2%)	0 (0.0%)	
	Early seroma	16 (3.6%)	2 (1.5%)	
	Rupture	0 (0.0%)	1 (0.8%)	
	Capsular contracture	14 (3.1%)	7 (5.3%)	
	Rippling	1 (0.2%)	0 (0.0%)	
	Shape deformity	23 (5.1%)	8 (6.1%)	
	Infection	7 (1.6%)	1 (0.8%)	
	Others	9 (2.0%)	6 (4.6%)	

**Abbreviations:** FU, follow-up.

Values are the number of cases with percentage.

Incidences of postoperative complications by the trade name of breast implants are represented in Table 7. This showed that there were significant differences in incidences of postoperative complications between the breast implants (P<0.05). Of note, incidences of CC were significantly higher in the patients receiving the Naturgel™ (4.0%, 8/199) or the BellaGel^®^ SmoothFine (4.1%, 10/245) as compared with the Eurosilicone Round Collection™ (0.0%, 0/32) or the Motiva Ergonomix™ (2.7%, 2/73) (P<0.05). Moreover, there was 1 case (0.2%) of rupture following the use of the Naturgel™. To avoid a comparison bias, we compared incidences of implant-related complications between the Naturgel™, the BellaGel^®^ SmoothFine and the Motiva Ergonomix™. This showed that incidences of rupture, CC, shape deformity and infection were calculated as 11.6% (23/199), 10.2% (25/245) and 9.6% (7/73) in the corresponding order. But these differences reached no statistical significance (P>0.05).

**Table 7:** Incidences of postoperative complications by the trade name of breast implants (n = 579).

Variables	Values								P
	Microtextured breast implants				Round smooth breast implants		Anatomical implants		
	Naturgel™ (n = 199)	BellaGel^®^ SmoothFine (n = 245)	Motiva Ergonomix™ (n = 73)	Eurosilicone Round Collection™ (n = 32)	Natrelle^®^ INSPIRA™ (n = 4)	Mentor^®^ MemoryGel Xtra (n = 13)	Microthane^®^ (n = 3)	Natrelle^®^ 410 (n = 10)	
**Total incidences**	38 (19.1%)	39 (15.9%)	13 (17.8%)	3 (9.4%)	2 (50.0%)	1 (7.7%)	3 (100%)	2 (20.0%)	<0.05*
Early hematoma	2 (1.0%)	3 (1.2%)	0 (0.0%)	0 (0.0%	0 (0.0%)	0 (0.0%)	0 (0.0%)	0 (0.0%)	
Delayed hematoma	1 (0.5%)	0 (0.0%)	0 (0.0%)	0 (0.0%	0 (0.0%)	0 (0.0%)	0 (0.0%)	0 (0.0%)	
Early seroma	10 (5.0%)	3 (1.2%)	3 (4.1%)	0 (0.0%	0 (0.0%)	0 (0.0%)	2 (66.7%)	0 (0.0%)	
Rupture	1 (0.5%)	0 (0.0%)	0 (0.0%)	0 (0.0%	0 (0.0%)	0 (0.0%)	0 (0.0%)	0 (0.0%)	
CC	8 (4.0%)	10 (4.1%)	2 (2.7%)	0 (0.0%	1 (25.0%)	0 (0.0%)	0 (0.0%)	0 (0.0%)	
Rippling	0 (0.0%)	0 (0.0%)	0 (0.0%)	0 (0.0%	0 (0.0%)	0 (0.0%)	0 (0.0%)	1 (10.0%)	
Shape deformity	10 (5.0%)	11 (4.5%)	5 (6.9%)	2 (6.3%)	0 (0.0%)	1 (7.7%)	1 (33.3%)	1 (10.0%)	
Infection	4 (2.0%)	4 (1.6%)	0 (0.0%)	0 (0.0%	0 (0.0%)	0 (0.0%)	0 (0.0%)	0 (0.0%)	
Others	2 (1.0%)	8 (3.3%)	3 (4.1%)	1 (3.1%)	1 (25.0%)	0 (0.0%)	0 (0.0%)	0 (0.0%)	

**Abbreviations:** CC, capsular contracture.

Values are the number of cases with percentage.

**Fig 7:**
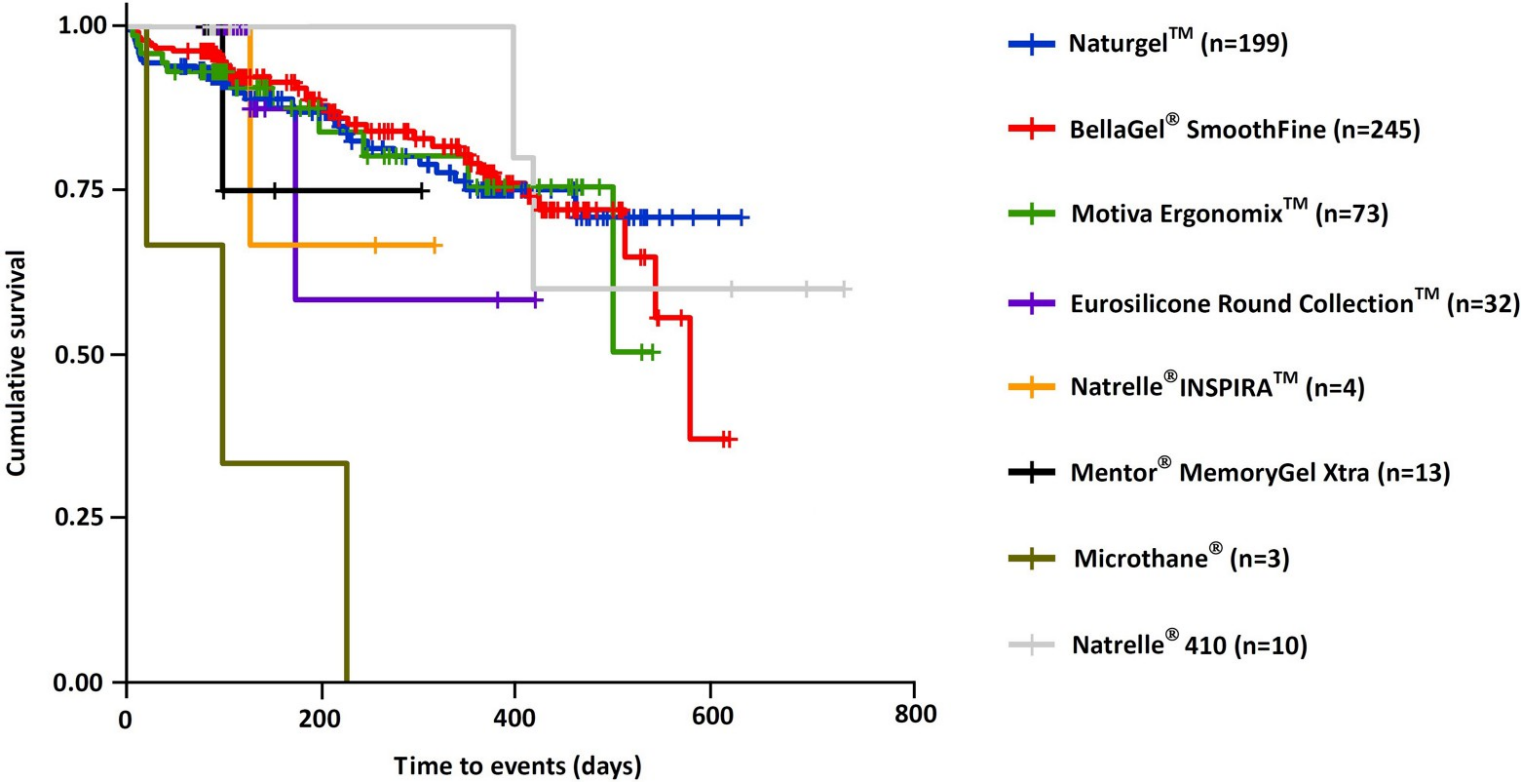
Cumulative complication-free survival by the trade name of breast implants.

**Fig 8:**
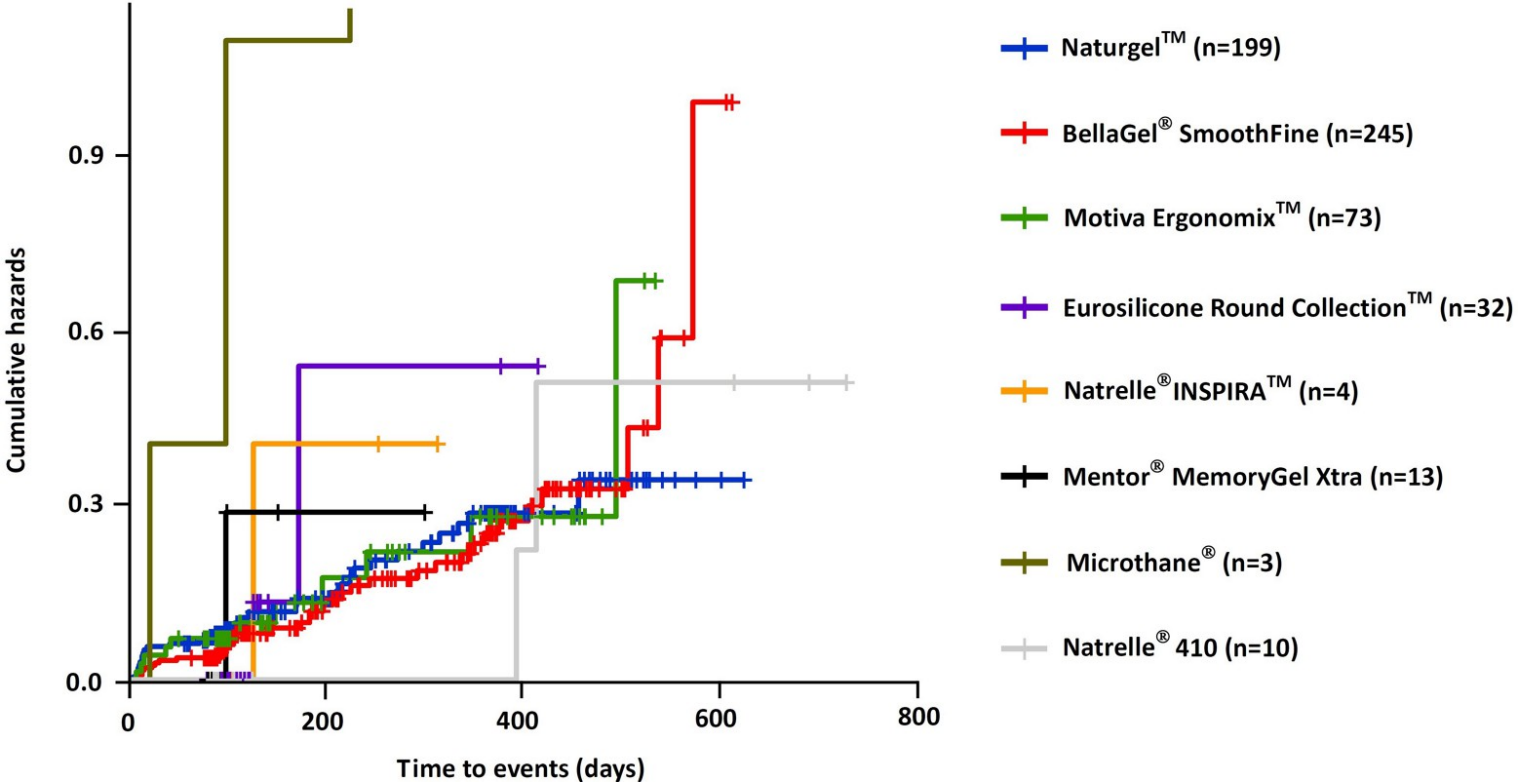
Cumulative complication-free hazards by the trade name of breast implants.

Cumulative complication-free survival period of the breast implant is shown in Table 8; the Natrelle^®^ 410 showed the longest survival (333.3±268.2 [141.5–525.1] days), followed by the BellaGel^®^ SmoothFine (209.2±154.2 [187.6–230.8] days), the Naturgel™ (209.1±150.8 [190.1–228.1] days), the Motiva Ergonomix™ (190.5±148.1 [155.9–225.1] days), the Natrelle^®^ INSPIRA™ (199.3±100.1 [39.9–358.6] days), the Motiva Ergonomix™ (190.5±148.1 [155.9–225.1] days), the Eurosilicone Round Collection™ (122.9±74.8 [95.9–149.8] days), the Microthane^®^ (114.3±102.5 [-140.2–368.9] days) and the Mentor^®^ MemoryGel™ Xtra (111.1±59.4 [75.2–147] days). In addition, cumulative complication-free survival rate is represented in Table 9. The Kaplan-Meier survival and hazards are shown in Figs 7 and 8, respectively.

**Table 8:** Cumulative complication-free survival period by the trade name of breast implants (n = 579).

		N	n	Censored value	TTEs (months)
**Total**		579	101	491 (84.8%)	201.4±151.6 (189–213.8)
	**Naturgel™**	199	38	167 (83.9%)	209.2±154.2 (187.6–230.8)
	**BellaGel**^**®**^ **SmoothFine**	245	39	209 (85.3%)	209.1±150.8 (190.1–228.1)
	**Motiva Ergonomix™**	73	13	62 (84.9%)	190.5±148.1 (155.9–225.1)
	**Eurosilicone Round Collection™**	32	3	30 (93.8%)	122.9±74.8 (95.9–149.8)
	**Natrelle**^**®**^ **INSPIRA™**	4	2	3 (75%)	199.3±100.1 (39.9–358.6)
	**Mentor**^**®**^ **MemoryGel Xtra**	13	1	12 (92.3%)	111.1±59.4 (75.2–147)
	**Microthane** ^**®**^	3	3	0 (0.0%)	114.3±102.5 (-140.2–368.9)
	**Natrelle**^**®**^ **410**	10	2	8 (80%)	333.3±268.2 (141.5–525.1)

**Note:** N, the total number of cases; n, incidences of postoperative complications.

**Abbreviations:** TTE, time-to-events

TTEs are expressed as mean±standard error with 95% confidence intervals.

**Table 9:** Cumulative hazards by the trade name of breast implants (n = 579).

	FU	N	n	Survival rate
**Naturgel™**	4	199	1	0.995±0.00501 (0.985–1.000)
	7	198	1	0.990±0.00707 (0.976–1.000)
	8	197	1	0.985±0.00864 (0.968–1.000)
	9	196	1	0.980±0.00995 (0.961–1.000)
	10	195	1	0.975±0.01109 (0.953–0.997)
	11	194	1	0.970±0.01212 (0.946–0.994)
	13	193	2	0.960±0.01392 (0.933–0.987)
	14	191	1	0.955±0.01473 (0.926–0.984)
	15	190	1	0.950±0.01549 (0.920–0.981)
	18	189	1	0.945±0.01620 (0.914–0.977)
	41	188	1	0.940±0.01687 (0.907–0.973)
	71	184	1	0.935±0.01754 (0.901–0.970)
	82	180	1	0.929±0.01819 (0.894–0.966)
	86	169	1	0.924±0.01890 (0.888–0.962)
	97	131	1	0.917±0.02003 (0.878–0.957)
	109	104	1	0.908±0.02169 (0.867–0.952)
	115	99	1	0.899±0.02333 (0.854–0.946)
	121	98	1	0.890±0.02483 (0.842–0.940)
	169	89	2	0.870±0.02801 (0.816–0.926)
	210	81	1	0.859±0.02965 (0.803–0.919)
	215	78	1	0.848±0.03125 (0.789–0.911)
	224	76	1	0.837±0.03277 (0.775–0.904)
	228	74	1	0.825±0.03422 (0.761–0.895)
	245	72	1	0.814±0.03561 (0.747–0.887)
	272	68	1	0.802±0.03705 (0.733–0.878)
	298	66	1	0.790±0.03843 (0.718–0.869)
	315	63	1	0.777±0.03981 (0.703–0.859)
	334	60	1	0.764±0.04120 (0.688–0.850)
	345	57	1	0.751±0.04260 (0.672–0.839)
	456	18	1	0.709±0.05712 (0.606–0.831)
**BellaGel**^**®**^ **SmoothFine**	8	245	2	0.992±0.00575 (0.981–1.000)
	13	243	2	0.984±0.00810 (0.968–1.000)
	15	241	1	0.980±0.00903 (0.962–0.997)
	23	240	1	0.976±0.00987 (0.956–0.995)
	26	239	1	0.971±0.01064 (0.951–0.993)
	30	238	1	0.967±0.01135 (0.945–0.990)
	48	237	1	0.963±0.01202 (0.940–0.987)
	90	210	1	0.959±0.01281 (0.934–0.984)
	93	185	1	0.953±0.01375 (0.927–0.981)
	94	183	1	0.948±0.01462 (0.920–0.977)
	96	174	1	0.943±0.01552 (0.913–0.974)
	100	154	1	0.937±0.01659 (0.905–0.970)
	105	144	1	0.930±0.01770 (0.896–0.966)
	108	136	1	0.923±0.01885 (0.887–0.961)
	141	113	1	0.915±0.02037 (0.876–0.956)
	174	105	1	0.906±0.02196 (0.864–0.951)
	183	103	2	0.889±0.02482 (0.842–0.939)
	197	95	1	0.880±0.02626 (0.830–0.933)
	206	94	1	0.870±0.02760 (0.818–0.926)
	216	90	1	0.860±0.02893 (0.806–0.919)
	225	88	1	0.851±0.03021 (0.794–0.912)
	244	83	1	0.840±0.03154 (0.781–0.905)
	292	74	1	0.829±0.03309 (0.767–0.897)
	311	71	1	0.817±0.03463 (0.752–0.888)
	338	64	1	0.805±0.03637 (0.736–0.879)
	350	60	1	0.791±0.03815 (0.720–0.870)
	360	57	1	0.777±0.03993 (0.703–0.860)
	375	48	1	0.761±0.04225 (0.683–0.849)
	405	39	1	0.742±0.04545 (0.658–0.836)
	419	35	1	0.720±0.04885 (0.631–0.823)
	506	10	1	0.648±0.08127 (0.507–0.829)
	537	7	1	0.556±0.11048 (0.376–0.821)
	572	3	1	0.371±0.16825 (0.152–0.902)
**Motiva Ergonomix™**	7	73	1	0.986±0.01360 (0.960–1.000)
	12	72	1	0.973±0.01910 (0.936–1.000)
	15	71	1	0.959±0.02320 (0.914–1.000)
	37	70	1	0.945±0.02660 (0.894–0.999)
	42	69	1	0.932±0.02960 (0.875–0.991)
	107	38	1	0.907±0.03760 (0.836–0.984)
	148	29	1	0.876±0.04760 (0.787–0.974)
	196	24	1	0.839±0.05790 (0.733–0.961)
	241	23	1	0.803±0.06590 (0.683–0.943)
	347	17	1	0.756±0.07710 (0.619–0.923)
	494	3	1	0.504±0.21200 (0.221–1.000)
**Eurosilicone Round Collection™**	126	8	1	0.875±0.11700 (0.673–1.000)
	172	3	1	0.583±0.25100 (0.251–1.000)
**Natrelle**^**®**^ **INSPIRA™**	126	3	1	0.667±0.27200 (0.300–1.000)
**Mentor**^**®**^ **MemoryGel Xtra**	98	4	1	0.750±0.21700 (0.426–1.000)
**Microthane** ^**®**^	21	3	1	0.667±0.27200 (0.300–1.000)
	98	2	1	0.333±0.27200 (0.067–1.000)
	224	1	1	0.000
**Natrelle**^**®**^ **410**	393	5	1	0.800±0.17900 (0.516–1.000)
	413	4	1	0.600±0.21900 (0.293–1.000)

**Note:** FU, follow-up; N, the total number of cases; n, incidences of postoperative complications.

Survival rates are expressed as mean±standard error with 95% confidence intervals.

### Results of a subgroup analysis

**Fig 9:**
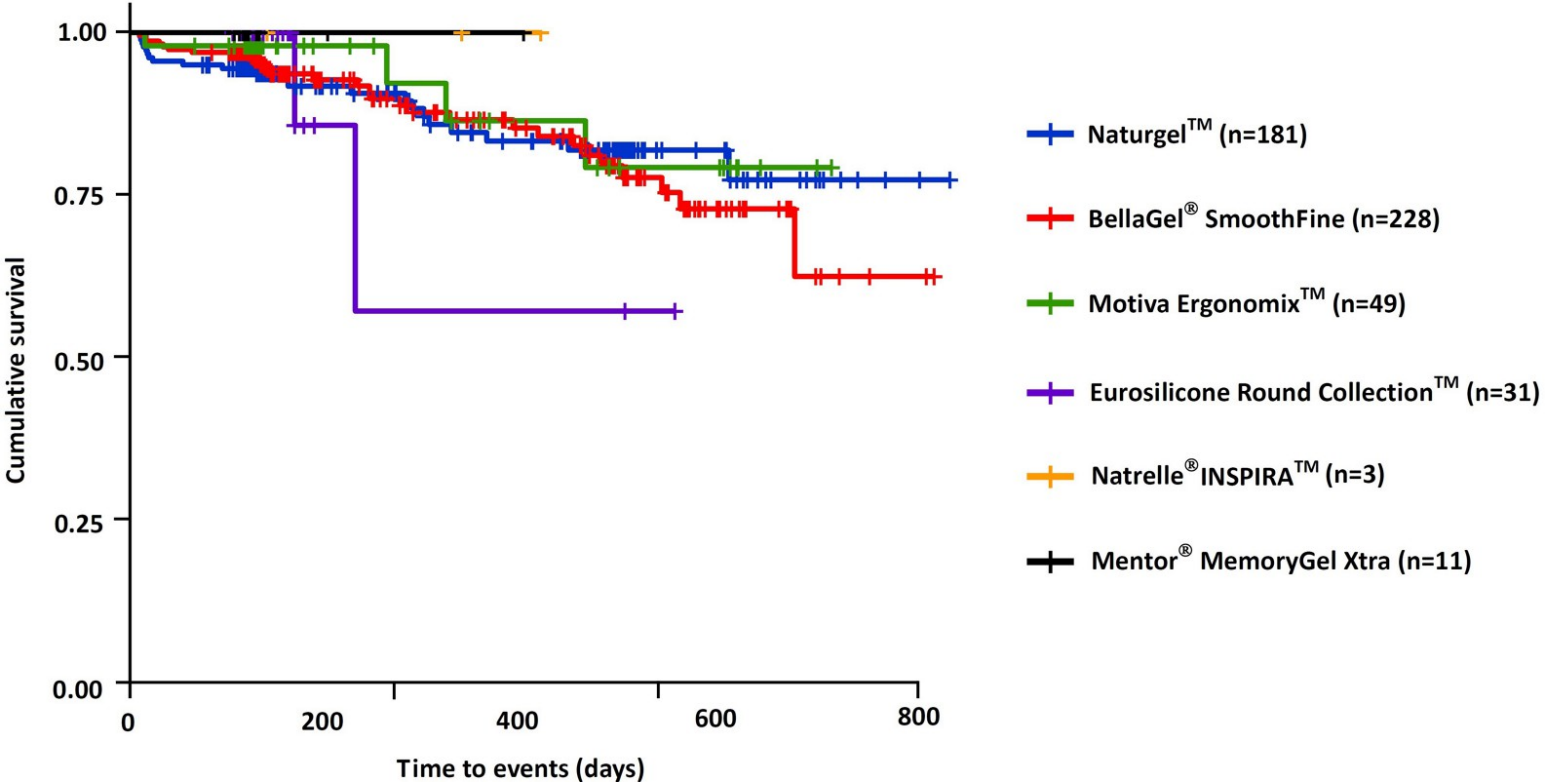
Cumulative complication-free survival by the trade name of breast implants on subgroup analysis.

**Fig 10:**
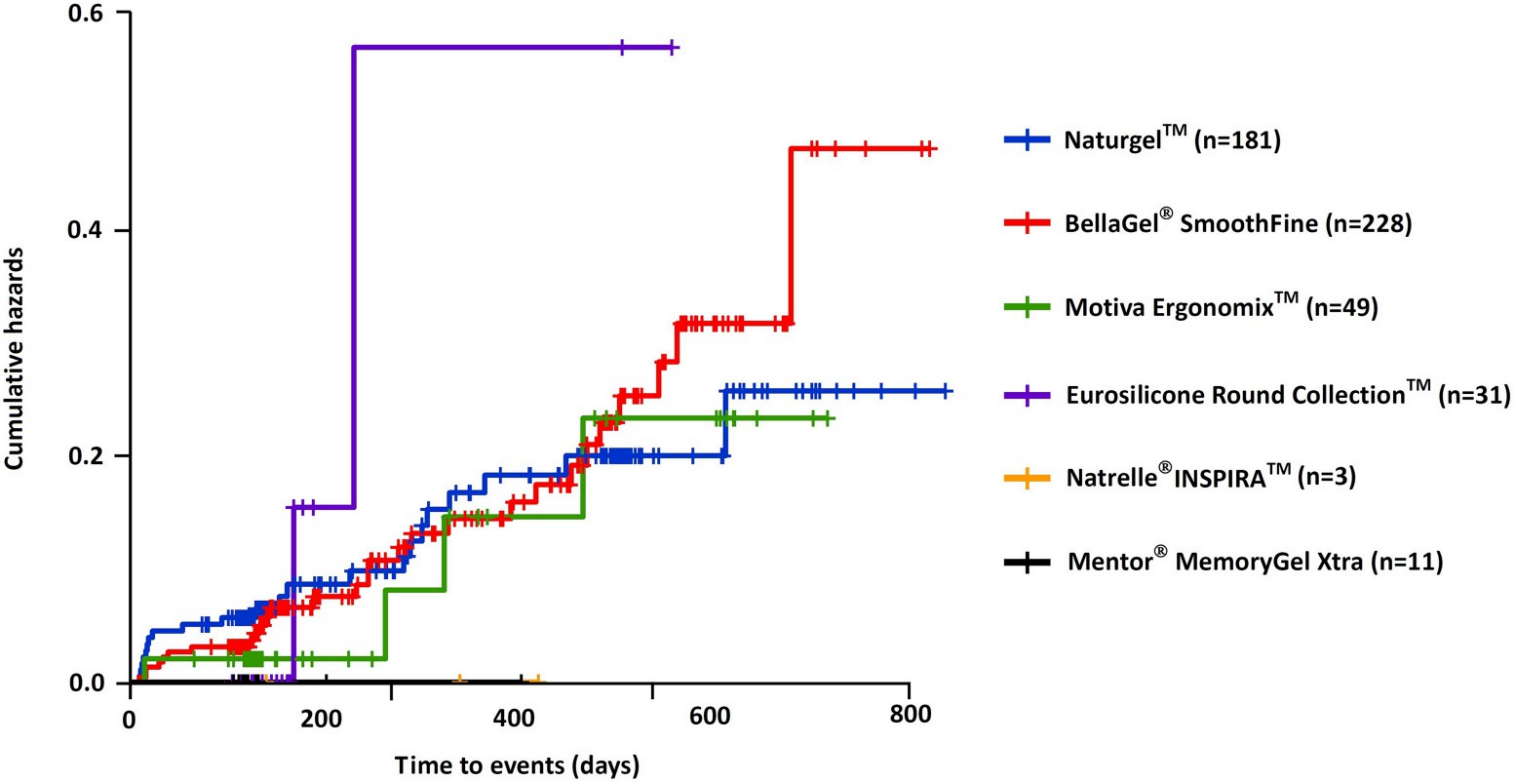
Cumulative complication-free hazards by the trade name of breast implants on subgroup analysis.

According to a subgroup analysis of incidences of postoperative complications, there were no significant differences in them between the breast implants (P>0.05) (Table 10). Moreover, the Natrelle^®^ INSPIRA™ showed the longest survival (223.7±107.1 [-42.3–489.6] days), followed by the BellaGel^®^ SmoothFine (218.0±156.2 [195.1–240.9] days), the Naturgel^TM^ (206.6±147.0 [195.1–240.9] days), the Motiva Ergonomix^TM^ (196.6±151.2 [153.2–240.1] days), the Eurosilicone Round Collection™ (122.6±76.0 [94.8–150.5] days) and the Mentor^®^ MemoryGel Xtra (114.4±64.5 [71.0–157.7] days) (Table 11). Furthermore, cumulative complication-free survival rate is represented in Table 12. The Kaplan-Meier survival and hazards are shown in Figs 9 and 10, respectively.

**Table 10:** Incidences of postoperative complications by the trade name of breast implants on subgroup analysis (n = 503).

Variables		Values						P
		Naturgel™ (n = 181)	BellaGel^®^ SmoothFine (n = 228)	Motiva Ergonomix™ (n = 49)	Eurosilicone Round Collection™ (n = 31)	Natrelle^®^ INSPIRA™ (n = 3)	Mentor^®^ MemoryGel Xtra (n = 11)	
**Total incidences**		27 (14.9%)	30 (13.2%)	4 (8.2%)	3 (9.7%)	0 (0.0%)	0 (0.0%)	>0.05
	Early hematoma	2 (1.1%)	1 (0.4%)	0 (0.0%)	0 (0.0%)	0 (0.0%)	0 (0.0%)	
	Delayed hematoma	1 (0.6%)	0 (0.0%)	0 (0.0%)	0 (0.0%)	0 (0.0%)	0 (0.0%)	
	Early seroma	7 (3.9%)	3 (1.3%)	1 (2.0%)	0 (0.0%)	0 (0.0%)	0 (0.0%)	
	Rupture	1 (0.6%)	0 (0.0%)	0 (0.0%)	0 (0.0%)	0 (0.0%)	0 (0.0%)	
	Capsular contracture	4 (2.2%)	8 (3.5%)	0 (0.0%)	0 (0.0%)	0 (0.0%)	0 (0.0%)	
	Shape deformity	6 (3.3%)	8 (3.5%)	2 (4.1%)	2 (6.5%)	0 (0.0%)	0 (0.0%)	
	Infection	4 (2.2%)	2 (0.9%)	0 (0.0%)	0 (0.0%)	0 (0.0%)	0 (0.0%)	
	Others	2 (1.1%)	8 (3.5%)	1 (2.0%)	1 (3.2%)	0 (0.0%)	0 (0.0%)	

Values are the number of cases with percentage.

**Table 11:** Cumulative complication-free survival period by the trade name of breast implants on subgroup analysis (n = 503).

		N	n	Censored value	TTEs (months)
**Total**		503	56	447 (88.9%)	202.7±147.8 (189.7–215.6)
	**Naturgel™**	181	27	159 (87.8%)	218.0±156.2 (195.1–240.9)
	**BellaGel**^**®**^ **SmoothFine**	228	30	200 (87.7%)	206.6±147.01 (187.4–225.8)
	**Motiva Ergonomix™**	49	4	45 (91.8%)	196.6±151.2 (153.2–240.1)
	**Eurosilicone Round Collection™**	31	3	29 (93.5%)	122.6±76.0 (94.8–150.5)
	**Natrelle**^**®**^ **INSPIRA™**	3	0	3 (100%)	223.7 ± 107.1 (-42.3–489.6)
	**Mentor**^**®**^ **MemoryGel Xtra**	11	0	11 (100%)	114.4±64.5 (71.0–157.7)

**Note:** N, the total number of cases; n, incidences of postoperative complications.

**Abbreviations:** TTE, time-to-events

TTEs are expressed as mean±standard error with 95% confidence intervals.

**Table 12:** Cumulative hazards by the trade name of breast implants on subgroup analysis (n = 503).

	FU	N	n	Survival rate
**Naturgel™**	8	181	1	0.994±0.00551 (0.984–1.000)
	9	180	1	0.989±0.00777 (0.974–1.000)
	10	179	1	0.983±0.00949 (0.965–1.000)
	11	178	1	0.978±0.01093 (0.957–1.000)
	13	177	1	0.972±0.01218 (0.949–0.997)
	14	176	1	0.967±0.01331 (0.941–0.993)
	15	175	1	0.961±0.01433 (0.934–0.990)
	18	174	1	0.956±0.01528 (0.926–0.986)
	41	173	1	0.950±0.01616 (0.919–0.982)
	71	169	1	0.945±0.01701 (0.912–0.979)
	97	124	1	0.937±0.01850 (0.901–0.974)
	115	95	1	0.927±0.02077 (0.887–0.969)
	121	94	1	0.917±0.02277 (0.874–0.963)
	169	85	1	0.907±0.02493 (0.859–0.957)
	210	78	1	0.895±0.02718 (0.843–0.950)
	215	75	1	0.883±0.02932 (0.827–0.942)
	224	73	1	0.871±0.03132 (0.812–0.934)
	228	71	1	0.859±0.03319 (0.796–0.926)
	245	69	1	0.846±0.03497 (0.780–0.918)
	272	65	1	0.833±0.03677 (0.764–0.908)
	334	59	1	0.819±0.03876 (0.746–0.899)
	456	18	1	0.774±0.05741 (0.669–0.895
**BellaGel**^**®**^ **SmoothFine**	8	228	1	0.996±0.00438 (0.987–1.000)
	13	227	2	0.987±0.00755 (0.972–1.000)
	23	225	1	0.982±0.00869 (0.966–1.000)
	26	224	1	0.978±0.00970 (0.959–0.997)
	30	223	1	0.974±0.01060 (0.953–0.995)
	48	222	1	0.969±0.01142 (0.947–0.992)
	93	171	1	0.964±0.01269 (0.939–0.989)
	96	162	1	0.958±0.01393 (0.931–0.985)
	100	143	1	0.951±0.01536 (0.921–0.982)
	105	133	1	0.944±0.01683 (0.911–0.977)
	108	125	1	0.936±0.01831 (0.901–0.973)
	141	104	1	0.927±0.02023 (0.888–0.968)
	174	96	1	0.918±0.02220 (0.875–0.962)
	183	94	2	0.898±0.02567 (0.849–0.950)
	206	87	1	0.888±0.02737 (0.836–0.943)
	216	83	1	0.877±0.02905 (0.822–0.936)
	244	77	1	0.866±0.03083 (0.807–0.928)
	292	69	1	0.853±0.03283 (0.791–0.920)
	311	66	1	0.840±0.03479 (0.775–0.911)
	338	59	1	0.826±0.03700 (0.757–0.902)
	350	55	1	0.811±0.03926 (0.738–0.892)
	360	52	1	0.795±0.04148 (0.718–0.881)
	375	43	1	0.777±0.04445 (0.694–0.869)
	405	34	1	0.754±0.04866 (0.664–0.856)
	419	30	1	0.729±0.05314 (0.632–0.841)
	506	7	1	0.625±0.10662 (0.447–0.873)
**Motiva Ergonomix™**	12	49	1	0.980±0.02020 (0.941–1.000)
	196	17	1	0.922±0.05900 (0.813–1.000)
	241	16	1	0.864±0.07860 (0.723–1.000)
	347	12	1	0.792±0.09970 (0.619–1.000)
**Eurosilicone Round Collection™**	126	7	1	0.857±0.13200 (0.633–1.000)
	172	3	1	0.571±0.24900 (0.243–1.000)

**Note:** FU, follow-up; N, the total number of cases; n, incidences of postoperative complications.

Survival rates are expressed as mean±standard error with 95% confidence intervals.

## Discussion

Since the introduction of a silicone gel-filled breast implant to augmentation mammaplasty, plastic surgeons and manufacturers of a device experience a transition from the previous generation of the device to the next generation of one. Thus, there has been an evolution in the breast implant design in an effort *not only* to improve aesthetic outcomes of an implant-based augmentation mammaplasty *but also* to minimize the occurrence of its complications [27]. Contemporary silicone gel-filled breast implants are characterized by increased surface roughness due to the modification to the shell surface. It remains problematic, however, patients receiving an implant-based augmentation mammaplasty are vulnerable to its long-term complications despite improvements in design and technology of a silicone gel-filled breast implant. Indeed, there is a variability in the mode of interaction between a device and the soft tissue depending on its surface characteristics [20]. Surface modification may result from previous *in vitro* and clinical studies showing positive correlations between surgical results and surface topography [28, 29]. Over the past decades, macrotexturization or microtexturization, such as the Mentor^®^ MemoryGel^TM^ SILTEX^®^ (Mentor Worldwide LLC) or the Natrelle^®^ BIOCELL^®^ (Allergan, Inc.), have been established as popular trends of implant technology [28, 30].

As nanotechnology has undergone technological advancements, a novel silicone gel-filled breast implant controlling cellular functions has emerged [31]. Moreover, it has also been used to improve the surface properties of a device. The Motiva Ergonomix™ VelvetSurface^®^ is equipped with 1,800–2,200 contact points of 40,000–100,000 nm depth, being much narrower as compared with a macrotextured device. Moreover, the Motiva Ergonomix™ SilkSurface^®^ is equipped with 49,000 contact points per 16,000 nm, being much smaller and shallower depressions as compared with a macrotextured or microtextured device [31]. Of note, according to the latest International Standards Organization (ISO) 14607:2018 definition, the Motiva Ergonomix™ SilkSurface^®^ is classified as the smooth breast implant [32]. Thus, manufacturers of a breast implant continue to use diverse methods for the development of a novel device for the purposes of stabilizing the device in the pocket by increasing the coefficient of friction or improving the integration of the device into the tissue [33]. It would therefore be mandatory to refine current best practices in an implant-based augmentation mammaplasty considering the unique features of diverse types of a device [34].

To date, the breast implant industry in Korea has experienced a transition from a textured device to a microtextured one. This is well illustrated in the current results showing that 94.8% of our clinical series of the patients received microtextured devices. Indeed, use of textured breast implants was banned by the KMFDS in Korea [11]. In other countries, plastic surgeons have moved towards smooth breast implants and manufacturers of a device and regulatory agencies discontinued the supply of textured devices in an effort to reduce risks of developing BIA-ALCL [35]. As shown in the current study, the Naturgel™, the BellaGel^®^ SmoothFine and the Motiva Ergonomix™ are representative popular brands of a microtextured breast implant. Of these, the BellaGel^®^ SmoothFine and the Motiva Ergonomix™ were compared for 1-year safety outcomes in a recent published study. This showed that CC occurred at a higher incidence in the patients receiving the BellaGel^®^ SmoothFine as compared with those receiving the Motiva Ergonomix™ (2.27% *vs*. 0.00%, P<0.05) [19]. This is in agreement with the current results; CC occurred at an incidence of 4.1% and 2.7% in the corresponding order.

According to a review of the previous published literatures, several studies have also been conducted to compare between the breast implants from different manufacturers in other countries [36–40]. These studies have reflected a transition from a round breast implant to an anatomical device or superiority of one anatomical device to another irrespective of whether they are retrospective or prospective in nature. We have efficiently used HRUS in performing a regular follow-up of our clinical series of the patients in a non-manufacturer-sponsored setting although the US FDA recommends that patients receiving a breast implant be further evaluation on magnetic resonance imaging scans at 3 years postoperatively and at a 2-year interval, and thereafter [17]. This is a different feature from previous comparative studies. The role of HRUS in examining the integrity and rotation of a breast implant has been of increasing interest [39, 41–48]. Moreover, its role has been expanded to manage patients who are suspected of having BIA-ALCL *as well as* to examine a breast mass [49, 50]. In this regard, plastic surgeons are required to make an HRUS-assisted approach to an implant-based augmentation mammaplasty. To do this, the following 2 matters should be considered: (1) Information about a breast implant (*e*.*g*., location, constituents, shell, shape and manufacturer) and (2) Possible occurrence of implant-related complications (*e*.*g*., folding with or without detachment, periprosthetic fluid collection, thickened capsule, rupture, capsular mass, malrotation of an anatomical device, upside-down rotation and foreign body reactions) [11].

**Fig 11:**
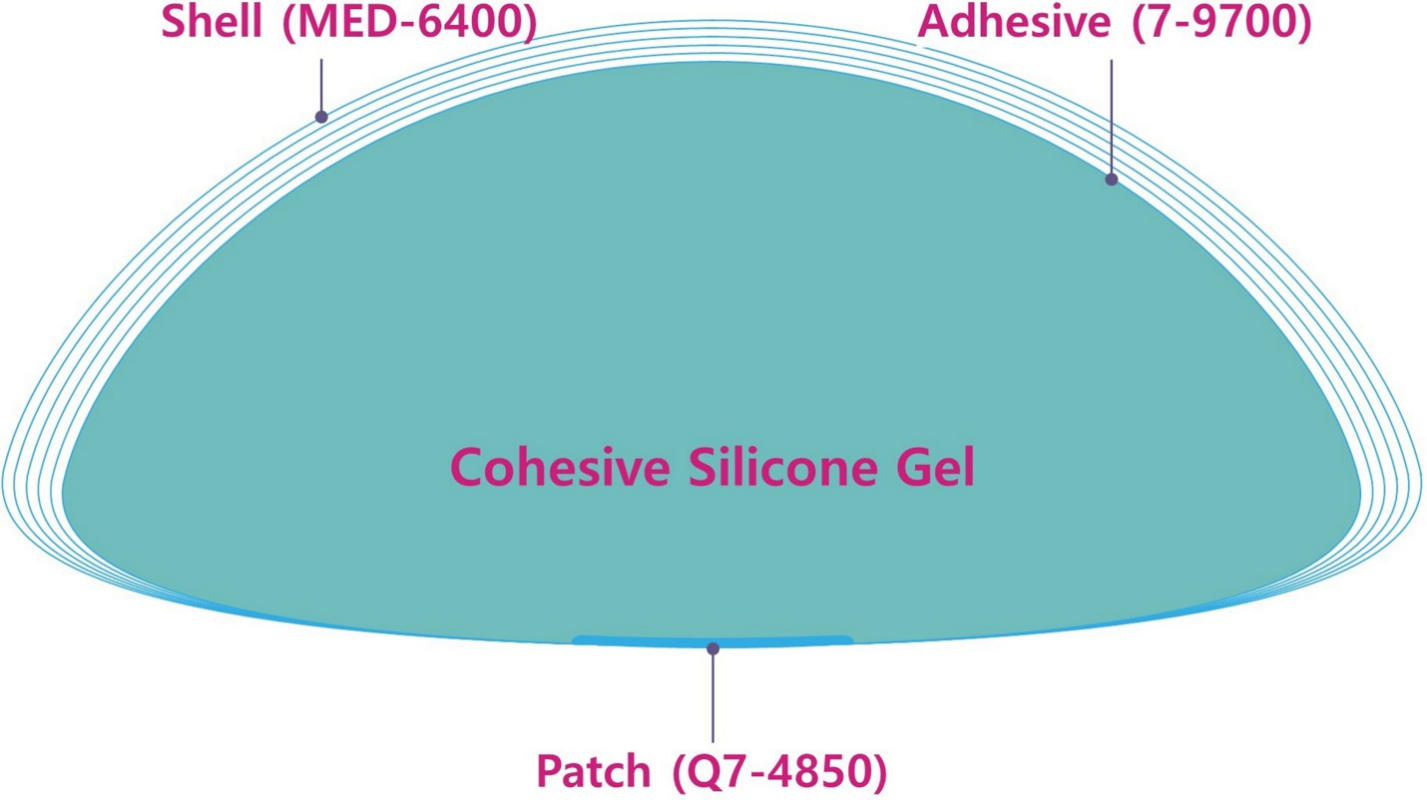
Unapproved materials used for manufacturing of the BellaGel^®^ implants.

Two-year safety outcomes of an implant-based augmentation mammaplasty using the BellaGel^®^ SmoothFine should be interpreted in the context of the first Korean case of a medical device fraud. Since 2009, the HansBiomed Co. Ltd. has illegally used unapproved substances, such as 7–9700 and Q7-4850, while manufacturing the BellaGel^®^ implants for the purposes of overcoming the detachment between the shell and gel of the device (Fig 11) [10].

*In vivo* use of 7–9700 is not permitted; it has been tested for cytotoxicity, skin irritation and skin sensitization, but no data has been obtained for mutagenicity/genotoxicity, pyrogenicity and system toxicity. In addition, Q7-4850 was also illegally used during manufacturing of the BellaGel^®^ implants including the BellaGel^®^ SmoothFine. This should be considered serious in that its *in vivo* use for >30 days is prohibited.

The Dow Corning^®^ Soft Skin Adhesives Parts A & B does not permit *in vivo* use of 7–9700 because its use has been permitted only for a wearable monitoring device or wound dressing. There is another problem that 7–9700 has been tested for cytotoxicity, skin irritation and skin sensitization without being tested mutagenicity/genotoxicity, pyrogenicity and system toxicity. Furthermore, *In vivo* use of Q7-4850 for >30 days is not also permitted [10]. Finally, the HansBiomed Co. Ltd. attempted to increase a soft feel of the BellaGel^®^ SmoothFine by deliberately modified its shell structure from 5- to 4-layered shell in violation of the regulatory requirement enforced by the KMFDS. Although the BellaGel^®^ SmoothFine with a 5-layered shell was approved by the KMFDS, the device with a 4-layered shell has also been circulated in the Korean market, for which Kim JH provided evidence on HRUS [11]. In November 13, 2020, the KMFDS initiated the mandatory recall of the BellaGel^®^ implants [10].

**Fig 12:**
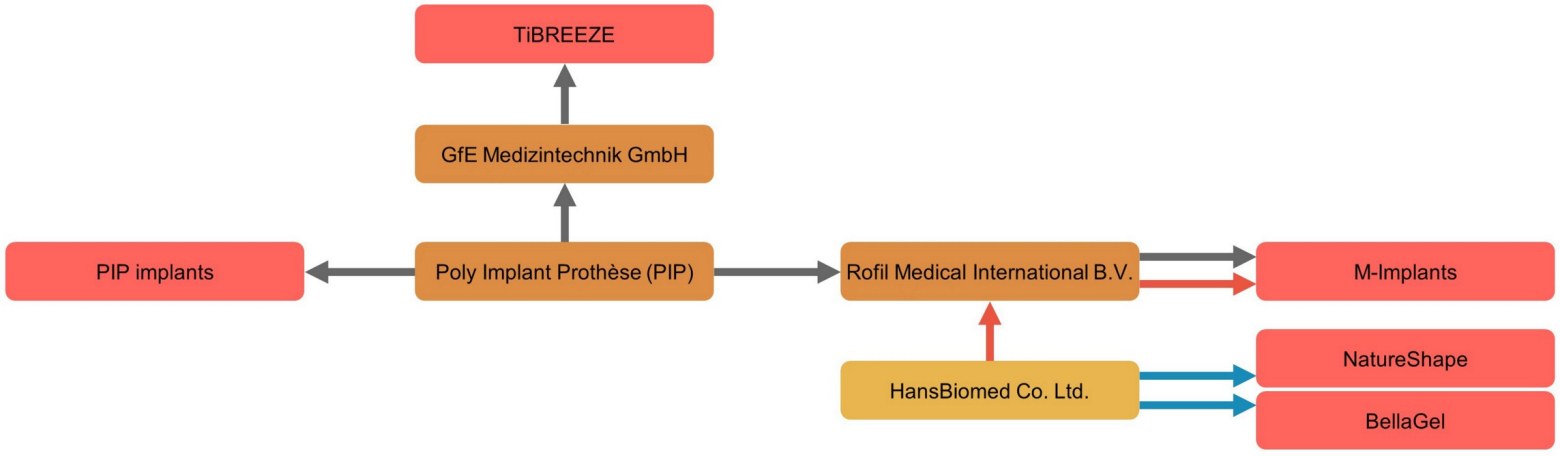
Previous involvement of the manufacturer of the BellaGel^®^ implants in the Poly Implant Prothèse (PIP) fraud.

Illegal use of unapproved substance and the deliberate modification of the shell structure during manufacturing of the BellaGel^®^ implants should be considered serious because the HansBiomed Co. Ltd. was previously involved in the PIP fraud by working as an original equipment manufacturer for the Rofil Medical International B.V. (Breda, Netherlands) that was a distributor of the M-Implants^®^ rebranded from the PIP (Fig 12) [10].

The manufacturer of the BellaGel^®^ implants was previously involved in the PIP fraud by working as an original equipment manufacturer for a distributor of the M-Implants^®^ rebranded from the PIP.

The BellaGel^®^ implants are non-FDA-approved products, although they have been described as a safe device according to manufacturer-sponsored studies [17, 51–54]. But their safety has become a mirage. Therefore, the safety of the BellaGel^®^ implants, including the BellaGel^®^ SmoothFine, should be stringently evaluated in an evidence-based manner.

There are no literatures indicating whether Korean plastic surgeons were aware of the manufacturer’s previous involvement in the PIP fraud; even the corresponding author of 2 manufacturer-sponsored studies was involved in the development and design of BellaGel^®^ implants, who is currently a non-executive medical director of the HansBiomed Co. Ltd. [10, 52, 54]. A 10-year prospective study was conducted to assess the efficacy and safety of the BellaGel^®^ implants in August 24, 2010, for which 6-year interim results have been published [53]. This strongly suggests that some Korean patients might have received the BellaGel^®^ implants that had been exported to Europe. Due to a lack of data about their composition that had not been characterized, such patients should be further evaluated for whether they had signs and symptoms indicating rupture or gel bleed. Moreover, it would also be mandatory to characterize the composition of the BellaGel^®^ implants on explantation study in patients with rupture of the device after receiving the BellaGel^®^ implants between August 24, 2010 and November 26, 2015 [10].

Two authors of 2 manufacturer-sponsored studies were reported to participate in the development of the BellaGel^®^ SmoothFine [11, 52, 54]. Choi MS, et al. conducted a multi-center, retrospective, preliminary observational study to assess the safety of BellaGel^®^ implants, including the BellaGel^®^ SmoothFine, in a consecutive series of 239 patients who received the devices at 3 hospitals between December 1, 2015 and January 31, 2018. This showed that 49.4% (118/239) of total patients received the BellaGel^®^ SmoothFine. Interestingly, the BellaGel^®^ SmoothFine was described as a nanotextured device rather than a microtextured one; one of its competitors, the Motiva Ergonomix™, has been commonly described as a breast implant with a nanotextured surface [32, 52, 55, 56]. Kang SH, et al. reported that 53.1% (530/621) of total patients received the BellaGel^®^ SmoothFine between November 27, 2015 and April 30, 2018 [54]. Taken together, it can be inferred that authors of 2 manufacturer-sponsored studies failed to disclose the accurate number of the patients receiving the 4-layered device between July 19, 2017 and April 30, 2018 [11, 52, 54].

To summarize, our results are as follows:

1. Overall, there were a total of 101 cases (17.4%) of postoperative complications; these include 31 cases (5.4%) of shape deformity, 21 cases (3.6%) of CC, 18 cases (3.1%) of early seroma, 8 cases (1.4%) of infection, 5 cases (0.9%) of early hematoma, 1 case (0.2%) of delayed hematoma, 1 case (0.2%) of rupture and 1 case (0.2%) of ripping. Moreover, there were also 15 cases (2.6%) of other complications.
2. Incidences of postoperative complications by the trade name include 19.1% (38/199), 15.9% (39/245), 17.8% (13/73), 9.4% (3/32), 50% (2/4), 7.7% (1/13), 100% (3/3) and 20% (2/10) in the patients receiving the Naturgel™, the BellaGel^®^ SmoothFine, the Motiva Ergonomix™, the Eurosilicone Round Collection™, the Natrelle^®^ INSPIRA™, the Natrelle^®^ 410, the Mentor^®^ MemoryGel Xtra or the Microthane^®^, respectively. These differences reached statistical significance (P = 0.034).
3. The Natrelle^®^ 410 showed the longest survival (333.3±268.2 [141.5–525.1] days), followed by the BellaGel^®^ SmoothFine (209.2±154.2 [187.6–230.8] days), the Naturgel™ (209.1±150.8 [190.1–228.1] days), the Motiva Ergonomix™ (190.5±148.1 [155.9–225.1] days), the Natrelle^®^ INSPIRA™ (199.3±100.1 [39.9–358.6] days), the Motiva Ergonomix™ (190.5±148.1 [155.9–225.1] days), the Eurosilicone Round Collection™ (122.9±74.8 [95.9–149.8] days), the Microthane^®^ (114.3±102.5 [-140.2–368.9] days) and the Mentor^®^ MemoryGel Xtra (111.1±59.4 [75.2–147] days).
4. According to a subgroup analysis of incidences of postoperative complications, there were no significant differences in them between the breast implants (P = 0.831). Moreover, the Natrelle^®^ INSPIRA™ showed the longest survival (223.7±107.1 [-42.3–489.6] days), followed by the BellaGel^®^ SmoothFine (218.0±156.2 [195.1–240.9] days), the Naturgel^TM^ (206.6±147.0 [195.1–240.9] days), the Motiva Ergonomix^TM^ (196.6±151.2 [153.2–240.1] days), the Eurosilicone Round Collection™ (122.6±76.0 [94.8–150.5] days) and the Mentor^®^ MemoryGel Xtra (114.4±64.5 [71.0–157.7] days).

But limitations of the current study are as follows: First, we conducted the current study under the retrospective design at local clinics in Korea. Therefore, the possibility of selection bias could not be completely ruled out. Second, we followed up our clinical series of the patients for short periods of time. Third, the number of the patients receiving the Natrelle^®^ INSPIRA^TM^ (n = 4), the Mentor^®^ MemoryGel Xtra (n = 13), the Microthane^®^ (n = 3) or the Natrelle^®^ 410 (n = 10) is much smaller as compared with other breast implants. These differences may arise from the popularity of a microtextured device in Korea, surgeons’ or patients’ preference and a variability in the surgical cost, ranging from USD 3,535.39 to USD 7,090.78. Therefore, the possibility of comparison bias could not be completely ruled out. Fourth, we failed to control other confounding factors that may affect incidences of CC. It would therefore be difficult to make a direct comparison between the breast implants from different manufacturers. To date, no prospective randomized controlled trials have been conducted to standardize such factors [57]. Fifth, we failed to exclude the patients receiving a breast implant *via* a trans-axillary incision or a peri-areolar incision because they account for 95.0% (550/579) of total patients. It has been reported that use of a trans-axillary incision or a peri-areolar incision is associated with a higher risk of CC as compared with an inframammary fold (IMF) incision [58, 59]. In Asian patients, an IMF incision is not frequently used because it leaves a notable scar; a trans-axillary incision or a peri-areolar incision are frequently used in Asian countries where it is not recommended that a scar be left on the breast [60, 61].

## Conclusions

Here, we describe preliminary 2-year safety outcomes of an implant-based augmentation mammaplasty using the BellaGel^®^ SmoothFine as compared with its competitors in Korean women. Our results showed that there were a total of 21 cases of CC. Of these, 10 cases (47.6%) occurred in the patients receiving the BellaGel^®^ SmoothFine. Interestingly, incidences of CC at 1, 2 and 3 years include 2.27% (6/264), 4.1% (10/245) and 8.8% (22/251), respectively; there were time-dependent increases in them [19, 62]. As Maxwell et al. previously reported, 6- and 10-year results obtained from the Natrelle^®^ 410 showed an approximately 1% annual increase in the incidence of CC of Baker grade III/ IV [22, 63]. According to previous published studies sponsored by the HansBiomed Co. Ltd., however, incidences of CC were reported to be 0.0% (0/239), 0.6% (1/78) and 1.6% (10/621) in patients receiving the BellaGel^®^ implants including the BellaGel^®^ SmoothFine [17, 52, 54]. Moreover, according to an experimental study sponsored by the HansBiomed Co. Ltd., comparing a risk of developing CC based on surface properties between the BellaGel^®^ implants, including the BellaGel^®^ SmoothFine, and the Motiva Ergonomix^TM^ SilkSurface, the BellaGel^®^ SmoothFine showed the lowest risk of developing CC [16]. Taken together, it can be inferred that published results of an industry-sponsored research should be interpreted with caution.

Despite a lack of statistical significance, our results showed that the Motiva Ergonomix™ showed the lowest incidences of implant-related complications of the 3 most popular brands of a silicone gel-filled breast implant. Considering both 1 case of rupture following the use of Naturgel^TM^ and the first Korean case of a medical device fraud involving the BellaGel^®^ SmoothFine, we recommend that Korean women receive the Motiva Ergonomix™ for an implant-based augmentation mammaplasty. This is in agreement with a recent paper published by Song KY, et al. concluding that the Motiva Ergonomix™ is a device of choice for Korean women in the context of the first Korean case of a medical device fraud [62]. But further large-scale, prospective, multi-center studies with a long period of follow-up are warranted to establish our results.

## Supporting information

S1 File (XLS)
